# Hemmule: A Novel Structure with the Properties of the Stem Cell Niche

**DOI:** 10.3390/ijms21020539

**Published:** 2020-01-14

**Authors:** Vitaly Vodyanoy, Oleg Pustovyy, Ludmila Globa, Randy J. Kulesza, Iryna Sorokulova

**Affiliations:** 1 Department Anatomy, Physiology and Pharmacology, College of Veterinary Medicine, Auburn, AL 36849, USA; pustoom@auburn.edu (O.P.); globalp@auburn.edu (L.G.); sorokib@auburn.edu (I.S.); 2 School of Kinesiology, Auburn University, Auburn, AL 36849, USA; 3 Department of Anatomy, Lake Erie College of Osteopathic Medicine, Erie, PA 16509, USA; rkulesza@lecom.edu

**Keywords:** vascular, node, mesenchymal, hematopoietic, endothelial, megakaryocyte, quiescence, bone marrow

## Abstract

Stem cells are nurtured and regulated by a specialized microenvironment known as stem cell niche. While the functions of the niches are well defined, their structure and location remain unclear. We have identified, in rat bone marrow, the seat of hematopoietic stem cells—extensively vascularized node-like compartments that fit the requirements for stem cell niche and that we called hemmules. Hemmules are round or oval structures of about one millimeter in diameter that are surrounded by a fine capsule, have afferent and efferent vessels, are filled with the extracellular matrix and mesenchymal, hematopoietic, endothelial stem cells, and contain cells of the megakaryocyte family, which are known for homeostatic quiescence and contribution to the bone marrow environment. We propose that hemmules are the long sought hematopoietic stem cell niches and that they are prototypical of stem cell niches in other organs.

## 1. Introduction

The bone marrow stem cells are very sensitive to their immediate environment [1]. Hence, it is important to nurture these cells in a specialized environment known as the stem cell niche [2,3,4]. Although the functional and, to some extent, structural and spatial properties of stem cell niches, have been characterized [5,6,7], the niche is yet to be clearly defined as a structural anatomical distinct unit. Several candidates have been proposed in this regard.

Blazsek and colleagues described a structure later termed hematon [8,9] that was proposed as the primary fundamental unit of hematopoiesis. The hematon is a spheroid (node) that measures around 100–500 micrometers in diameter. The spheroid contains adipocytes/preadipocytes cells, mesenchymal cells, reticular cells, and macrophages. Additionally, the hematon is comprised of myeloid, erythroid, and megakaryocyte progenitor cells [10]. The procedure of hematon separation from the adult mouse femoral bone includes mechanical fractionation of bone marrow that involves dilacerations by scalpels and the removal of hematons by aspiration [11]. This procedure makes it difficult to determine the original location of hematons and their potential interconnections, thus questioning whether the hematon is indeed a stem cell niche.

Similar by size and composition, potential stem cell niches have been observed in human fetal bone marrow by Peault and colleagues [12], who demonstrated that endogenous marrow hematopoiesis started within the 11th week of development in specialized chambers, known as “primary loggetes,” structures organized by vascular cells. The subsequent hematopoiesis grew significantly within rapidly expanding logettes that were densely populated with cells of the erythroid and granulocytic series by week 15. This was a strong indicator that the vascular chambers contain the earliest colonizing hematopoietic stem cells [12,13,14]. However, loggetes initially become discernible in the later stages of development and then were no longer visible [12], making them unlikely candidates for adult stem cell niches.

Using immunostaining of bone sections, laser scanning confocal microscopy, and 3D tissue reconstruction, Adams and colleagues [15] demonstrated that the periphery of the adult bone marrow cavity in mice contained compartments with special properties. These structures, which were named “hemosphere” contained stem cells of the hematopoietic lineage. However, hemosphere cannot function alone as a stem cell niche without being integrated with outside sinusoidal endothelial cells and osteoblasts [16].

Nardi and colleagues isolated “three dimensional cell complexes” in the bone marrow from the distal end of mouse tibia and femur bones by centrifugation and filtration [17]. These complexes contained mesenchymal and hematopoietic stem cells, but whether these structures exist in the bone marrow or are a result of its fractionation is yet to be resolved.

Roche and colleagues [18] used synchrotron radiation microtomography to identify in the adult mouse femur and tibia bones what they call vascular sinusoid lobules and suggested that these lobules may serve as stem cell niche; however, their cell composition remains unknown.

A niche is defined as a cellular and molecular microenvironment that regulates the quiescence, proliferation and differentiation of stem cells. The stem cells in the niche have ability to self-renew and differentiate into all cells that need replacement during the lifetime [2,3,4].

Confined geometry allows a niche to function better [19,20]. Determining the architecture of niche stem cells has been hindered by the challenges in maintaining structural stability when sectioning bone, coupled with the difficulty of identifying cells immunochemically [21].

Here, we report a node-like structure from the bone medullary cavity that we termed hemmule and propose that this structure is the long-sought stem cell niche.

## 2. Results

Here, we will describe the morphology and fine structure of hemmule, the nature of hemmule vessels, as well as the hemmule cell composition. We will demonstrate that hemmule structural and cellular properties are very distinct from those of the control samples of bone marrow, lymph node, and blood vessel, and we will examine whether or not the hemmule properties meet the Schofield criteria of a stem cell niche [22]: (1) a distinct anatomic position, (2) a space where stem cells can be maintained and replicate, (3) a place where differentiation was inhibited, and (4) a restricted space that limited the quantities of stem cells.

### 2.1. Morphology and Fine Structure of Hemmule

**Figure 1:**
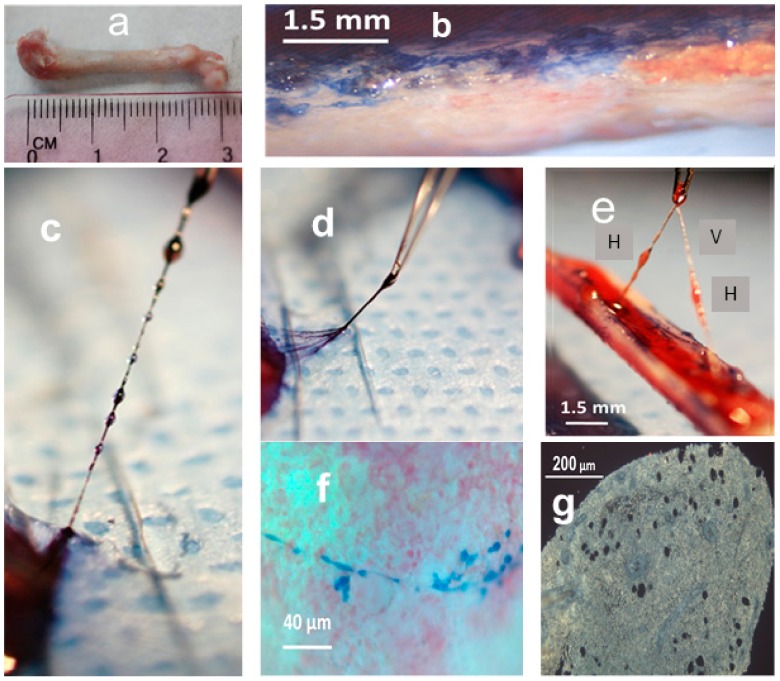
The hemmule and vessels taken from the freshly split rat femur bone. (**a**) Femur bone. (**b**) A split bone exposes a bone marrow stained by the trypan blue. (**c**) A series of hemmules are connected to the vessel. The lower hemmule is attached to the end hemmule inside the bone marrow. The hemmules and vessels are stained with trypan blue. The distance between the centers of the background dots is 1.5 mm. (**d**) The efferent vessel of the lower end hemmule is composed of multiple subvessels. (**e**) These hemmules are extracted from the bone marrow, while both ends of the vessel are attached to the end hemmules in the bone marrow. H—hemmule, V—vessel. (**f**) A thin branched vessel in the non-fixed bone marrow is stained by trypan blue. (**g**) A slice of the fixed hemmule is extracted from the bone marrow. Openings are voids of large size cells.

The hemmules were extracted from the split in halves femur rat bone, as described in the Material and Methods section (Figure 1). While replicating the Blazsek hematon procedure [11], we noticed that peculiar nodes can be extracted from the split bone without aspiration of bone marrow. A gentle application of 0.2% of the trypan blue to the surface of bone marrow revealed branching blue tracks. The lifting of the stained vessel exposed nodes (hemmules) that looked like beads. The vessel that was lifted in the nearby space of an epiphysis contains a distal hemmule with efferent vessel branching in several thin sub-vessels (Figure 1d). Figure 1e illustrates a vessel (V) with hemmules (H) lifted from bone marrow. It is possible to lift the hemmules with outside vessels using surgical tweezers without any noticeable resistance. It seems as though the hemmule and vessels are not fixed (or only weakly fixed) to the BM extracellular matrix. Other BM vessels—blood and lymphatic—cannot be extracted so effortlessly. Typically, the lifted hemmules have one afferent and one efferent vessel that comprises of several subvessels. The hemmules are oval with the length/width ration of 2–2.5.

The size of these hemmules varies significantly from 0.3–0.6 mm on the short axis and 0.6–1.5 mm on the long axis. When a hemmule is lifted from BM without staining with trypan blue, it appears slightly pinkish white, while the vessels are colorless. The surface of the hemmules is smooth and shiny. They are soft to touch and elastic.

**Figure 2:**
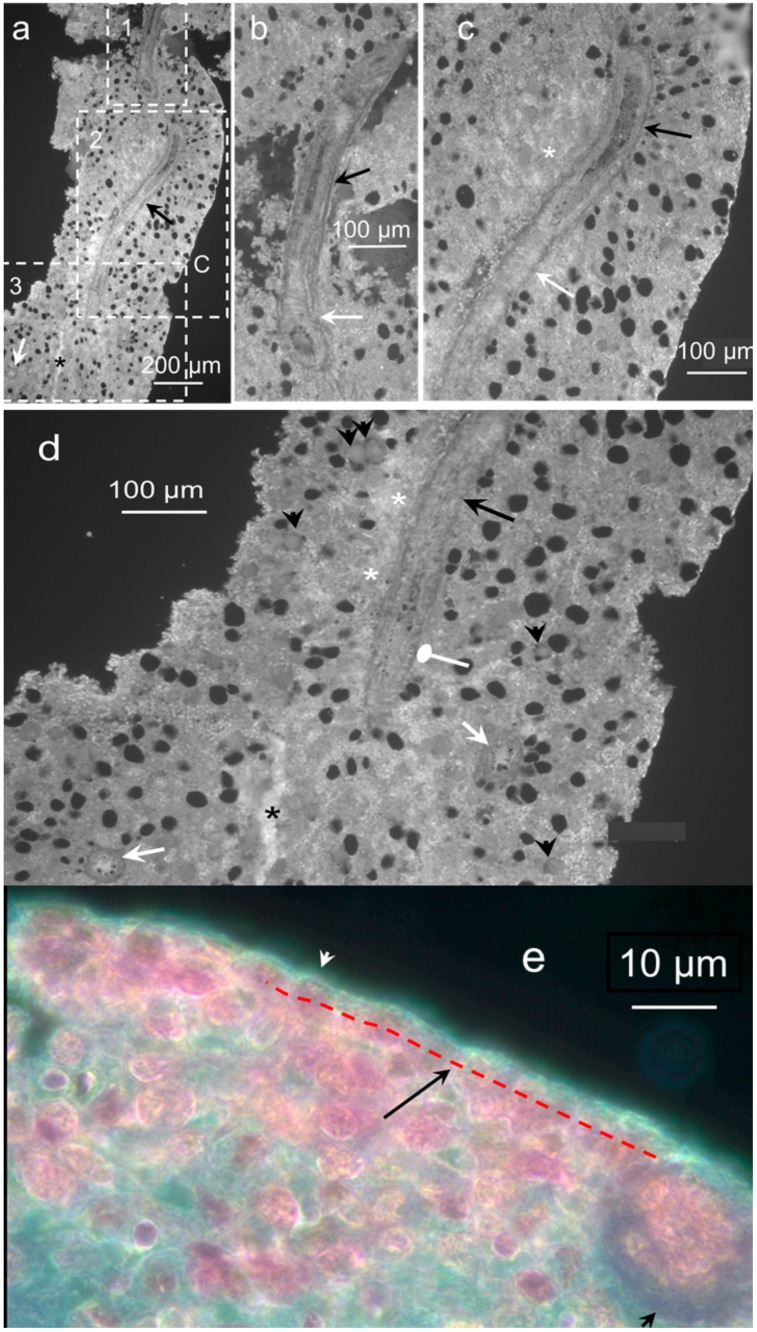
Longitudinal section of hemmule. A section of 6 μm from the rat femur bone marrow. (**a**) The section shows a portion of the central vessel passing in the longitudinal direction (black arrow). A smaller longitudinal vessel is labeled using an asterisk. The lumen of one transversal vessel is identified by a white arrow. Letter C depicts a fragment of the capsule. The section is divided by three areas of interest, marked as 1, 2, and 3 and shown in panels (**b**–**d**), respectively. (**b**) The magnified area 1 from panel (**a**) depicts a top part of the central vessel. The lower portion of the vessel (white arrow) illustrates the lumen with thick walls. A higher part of the vessel (black arrow) cuts open to expose a central duct with thick walls. (**c**) The magnified area 2 from panel (**a**). A white arrow reveals part of the vessel with a longitudinal ribbed wall surface. A black arrow points to the cut open vessel with thick walls. A white asterisk denotes an area with a high concentration of small vessels. (**d**) The magnified area 3 from panel (**a**). The central longitudinal vessel (long black arrow) is shown to be cut open (round head white arrow) before going down transversely into the slide. Two more large transverse vessels lumens are visible in this section (white arrows). The slice is highly vascular, depicting the density of small vessels (or channels) in several parts of the slide and exemplified by the areas marked by white asterisks. A relatively large vessel or a channel is labeled using a black asterisk. The entire area of the slice is covered with round holes, which shows the footprints of missing large size cells. Some of these cells are in close proximity to the holes (short black arrows). (**e**) The magnified portion of hemmule. White arrowhead shows the capsule. Black arrow illustrates the layer of cell around the hemmule border (red dotted line). Black arrowhead shows a cross-section of the transversal vessel. Sections a-d are not stained; section e is stained by Hematoxylin and eosin stain (H&E).

To characterize the structure of the hemmules, we used a high-resolution microscopy of the hemmule thin sections. A typical longitudinal section of hemmule is illustrated in Figure 2.

The hemmule of the oval shape of 600 × 1600 μm is surrounded by a fibrous capsule. In addition, the central vessel passing through the longitudinal direction almost through the entire hemmule is visible on the surface of the slide, along with many smaller longitudinal vessels and channels. The longitudinal and transversal vessels create a single vascular system within the hemmule. The central vessel of about 50 μm in diameter contains the lumen with thick walls girded by surrounding rib-like structures. The extracellular matrix creates seemingly sponge-like tissue to provide small internal channels, which enables cells and other constituents to travel and interact within the hemmule. The channel is distinguished from the vessel by the smaller size and absence of a wall. This is illustrated in Figure 2d, where black and white arrow show vessels, but a channel is indicated by an asterisk.

**Figure 3:**
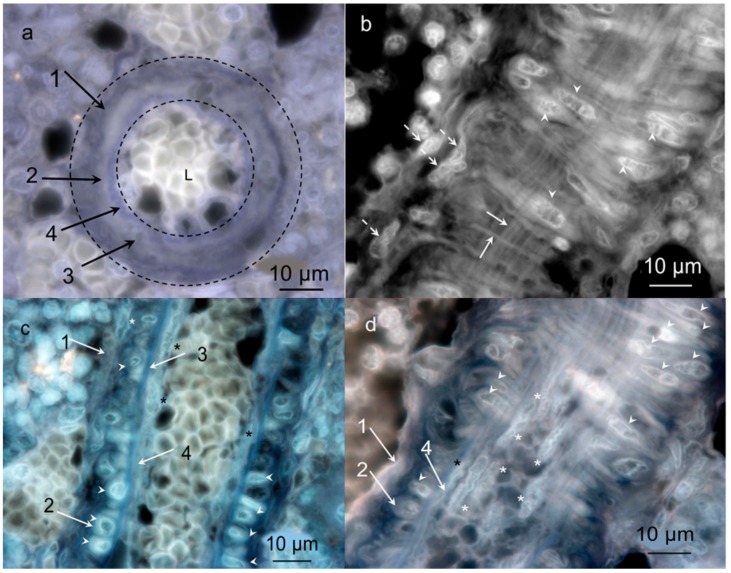
High magnification darkfield images of the vessel inside the hemmule. (**a**) Transversal section of the hemmule vessel. The wall of the vessels is composed of several optically distinguished Layers 1–4; L—lumen. (**b**) The longitudinal section of the vessel: arrowheads—vascular smooth muscle cells comprised the thick fibers-like loops (Layer 2), solid arrows—longitudinal thin fiber treads, and transversal fiber, respectively, dotted arrows—longitudinal fibroblastic cells and their processes belonging to the vessel external Layer 1. (**c**) The longitudinal section cuts the middle portion of the vessel. White arrows-layers 1–4, arrowheads—cross-sections of vascular smooth muscle cells of Layer 2, white asterisk—represent a fibroblastic cell of the Layer 1, and black asterisks denote endothelial cells of Layer 4. (**d**) The longitudinal section cuts the vessel wall in the vicinity of Layer 3, the subendothelial layer, primarily comprising of finely dispersed smooth muscle cells and bundles of fibrils. White asterisks—dispersed smooth muscle cells of Layer 3, arrows—Layers 1, 2, and 4, respectively, arrowheads—cross-sections of vascular smooth muscle cells of Layer 2, black asterisk—endothelial cell in Layer 4. No sections are stained. Pseudo color is caused by auto fluorescence.

We used high magnification darkfield microscopy and optical slicing to reveal the vessel inside the hemmule (Figure 3, Figures S1 and S2).

Figure 3a presents the transversal section of the hemmule vessel. Panels (b–d) of Figure 3 represent three consecutive longitudinal optical slices in close proximity to the top portion of the vessel (b), through the middle of the vessel (c), and in close proximity to the lower part of the vessel (d). Meanwhile the wall of the vessels comprises of several optically distinguished layers: the outmost external Layer 1 (cells are positioned along the vessel), the middle Layer 2 (transversal cells), the second middle layer (cells are positioned longitudinally), and the internal Layer 4 are composed of longitudinal cells. The characteristic features of Layer 2 are vining around the vessel thick fiber-like loops and longitudinal thin fiber treads that stretch parallel to the vessel in its entirety. This vessel is filled with small 3–4 µm cells.

The above morphological analyses reveal a specific anatomical place of hemmule in the bone marrow and determine the volume-restricted node-like unit surrounded by fibrous capsule, which reveals the vascular nature of hemmule and the four-layer structure of the hemmule vessels.

These properties meet the Schofield requirement of a distinct anatomic position and fulfill the criterion of a restricted space that limited the number of stem cells.

### 2.2. Nature of Hemmule Vessels

#### 2.2.1. Comparison of Hemmule Vessels with Blood and Lymphatic Vessels

**Figure 4:**
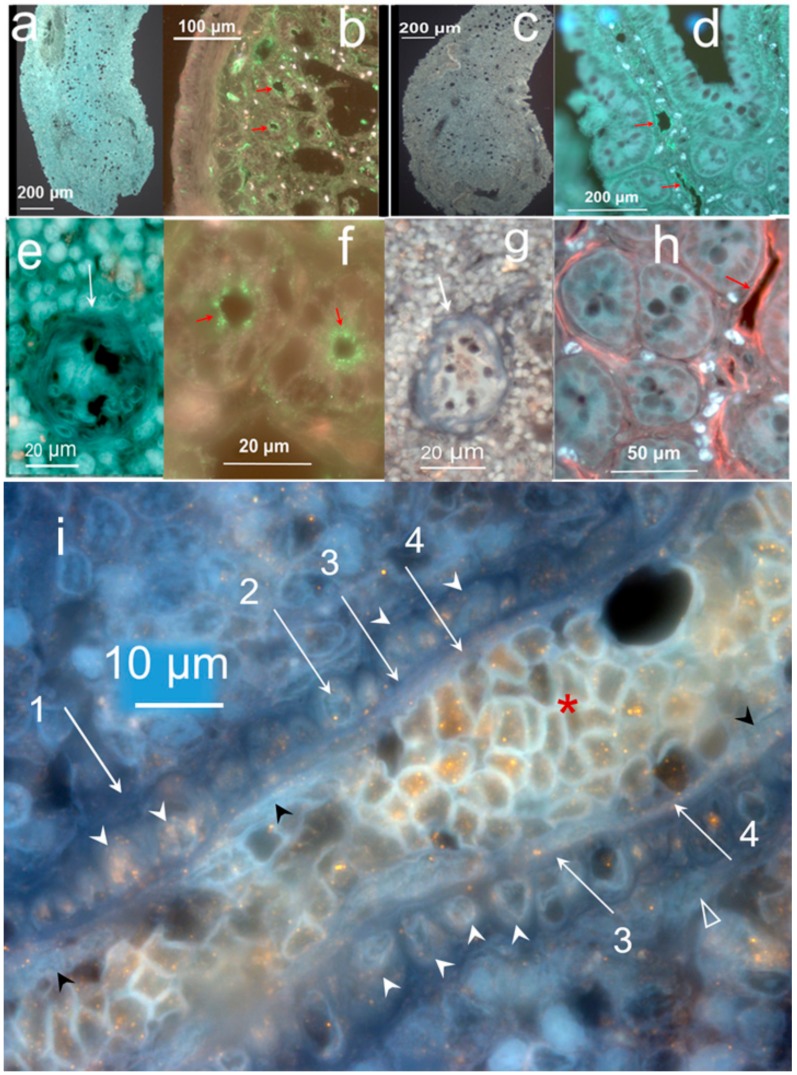
Hemmule does not interact with RECA-1 antibody (blood vessel endothelial cell marker) and with LYVE-1 (lymphatic vessel endothelial cell and hyaluronan receptor marker). Immunofluorescence staining of hemmule and small intestine. (**a**,**e**) Fluorescence images of a rat hemmule cross section treated with RECA-1 polyclonal antibodies and Goat Anti-Rabbit IgG as secondary antibodies visualized by Alexa 488. The white arrow in panel **e** shows lumens of the hemmule vessel. (**b**,**f**). Fluorescence image of a cross-section of small intestine is labeled with RECA-1 antibodies (positive control). The vascular profiles are located on the luminal aspect of the inner circular. Red arrows denote endothelial lining of blood vessels (green). (**c**,**g**). Fluorescence images of a rat hemmule cross-section treated with LYVE-1 polyclonal antibodies and Goat Anti-Rabbit IgG as secondary antibodies and visualized by Alexa 488. The white arrow in g indicates lumen of the hemmule vessels. (**d**,**h**) Fluorescence image of a cross-section of small intestine labeled with LYVE 1 antibodies (positive control), visualized with Alexa 488 (**d**) and Alexa 555 (**h**). Red srrows show endothelial lining of lymphatic vessels. (**i**) Fluorescence images of cells within the longitudinal cross-sections of hemmule are immunostained with the anti-LYVE-1 antibody. The longitudinal section cuts the middle portion of the vessel. Red star—the intra-vessel cells stained by the anti-LYVE-1 antibody; white arrows—Layers 1–4; white arrowheads—cross-sections of vascular smooth muscle cells of Layer 2; black arrowhead—endothelial cells of the Layer 4; open triangle—fibroblastic cell of Layer 1. Layer 4 of endothelial cells is not stained by anti-LYVE-1 antibody.

In order to examine whether the vessels in the hemmule belong to blood or lymphatics vasculatures, we visualized blood vessel and lymphatic endothelial cell markers (Figure 4).

Figure 4 illustrates both low and high magnification images of the hemmule besides the small intestine sections treated with blood vessel endothelial and lymphatic cell markers. These images indicate that while both cell markers do not stain the hemmule sections, they do stain the positive control cells of the small intestine. The secondary antibodies did not bind the hemmule cells (negative control, Figure 4c,g) in the absence of primary markers. At the single resolution (Figure 4i), cells inside the hemmule vessel are stained using LYVE-1 (lymphatic endothelial cell marker), but that is not the case with the most internal layer of endothelial cells. 

**Figure 5:**
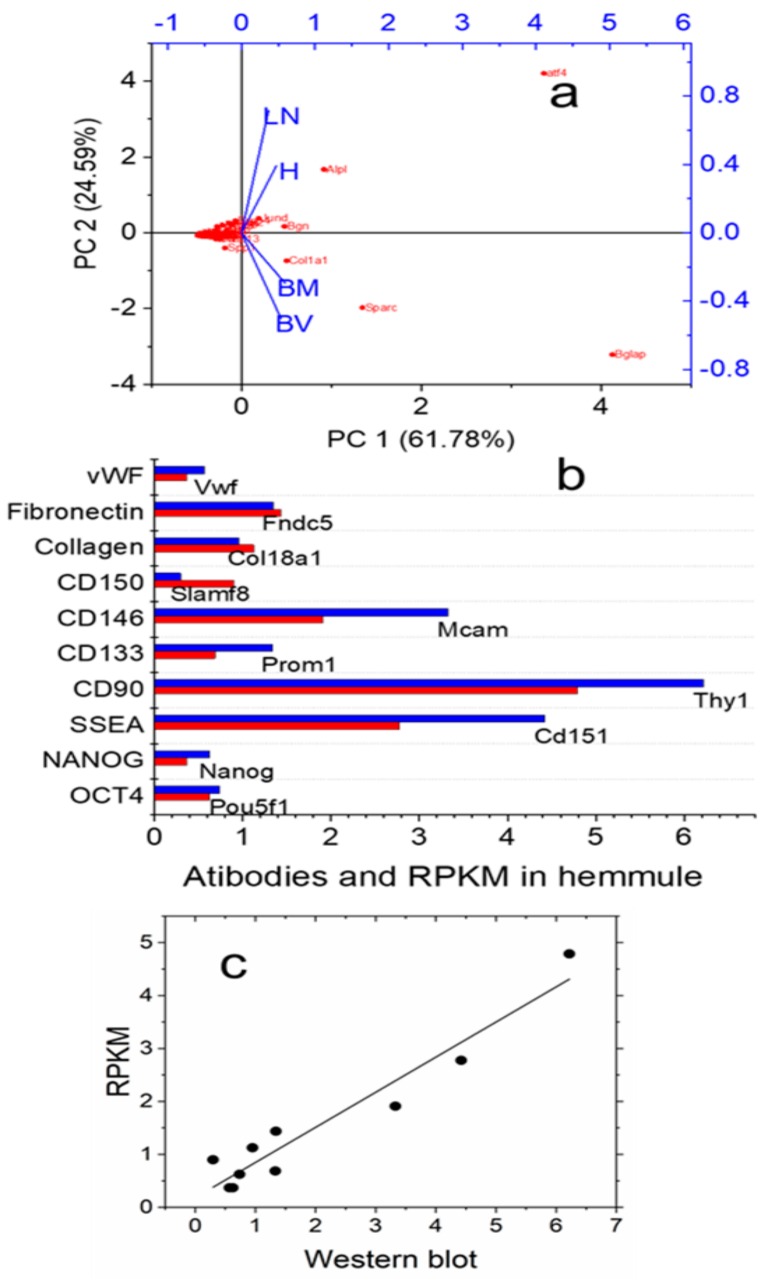
Principal component analyses (PCA), Western blot, and RNA-Seq. Protein expression obtained by Western blot and reads assigned per kilobase per million mapped reads (RPKM) correlation. (**a**) Biplot graph of the principal component analysis using gene expression (RPKM values) for the selected niche genes, hematopoietic progenitor, mesenchymal, and osteogenic genes. H, BV, BM, and LN are hemmule, blood vessel, bone narrow, and lymph node, respectively. (**b**) The lineup of relative levels of protein expressions (blue) and normalized RNA reads, RPKM (red). (**c**) The linear correlation between RPKM and relative levels of proteins. Pearson’s r = 0.96; Adj. R-square = 0.91.

Figure 5 characterizes hemmules by PCA analyses, Western blot, and RNA-Seq Western blot, RNA-Seq.

A very small level of RECA-1 antibody in hemmule indicates a negligible level of endothelial cells inherent in blood vasculature. In comparison, the RECA-1 is of relatively high concentration in BM and denotes the presence of endothelial cells characteristic of blood vessels. This result is in consonance with our observation made using fluorescent microscopy that the RECA-1 cells are absent in hemmule (Figure 4a,e). The high-level LYVE-1 signal could justify the large concentration of lymphatic endothelial cells in hemmule or other cells that contain an abundance of hyaluronan receptors. Evidence suggests that LYVE-1 labels cells in the other than lymphatic vasculature rich in hyaluronan receptors [23,24,25]. This finding implies that LYVE-1 binds hyaluronan receptors of non-lymphatic vasculature in hemmule. Importantly, this conclusion is consistent with the absence of LYVE-1 antibody expression in the wall of hemmule vessel (Figure 4e). The assumption becomes even more convincing in the high-resolution fluorescent image of a longitudinal section of hemmule vessel, which shows that LYVE-1 is not expressed by the endothelial cells of the vessel but binds cells inside the vessel (Figure 4i). Based on these experiments, we can conclude that hemmule vessels do not belong to blood or lymph vasculatures.

#### 2.2.2. RNA Expression Patterns in the Hemmule, Bone Marrow, Lymph Node, and Blood Vessel

RNA-Seq analysis was carried out to compare RNA expression pattern of the hemmule with those of other tissues. Here, 519 genes were identified for samples of hemmule, blood vessel, BM, and lymph node. Three sets of gene lists were generated by comparing the hemmule group with three other groups, including blood vessel (H-vs.-BV), bone marrow (H-vs.-BM), and lymph node (H-vs.-LN) groups. Each comparison group is depicted by a list of representative genes for the samples blood vessel, bone marrow, and lymph node. The differences in gene expression between the samples can be directly evaluated by comparing RPKM values. The representative genes for each sample (for each comparison group) have significantly different RPKM values. Based on RNA analysis and a comparison between hemmule and bone marrow samples, it can be concluded that 17 genes have four-fold difference in RPKM at a 95% confidence level, whereas six genes reveal an eight-fold difference in RPKM at a confidence level of 98%. This indicates that the hemmule is very different from the bone marrow. The comparison between the hemmule and the lymph node reveals 147 genes with four-fold difference at a confidence level of 95% and 32 genes with eight-fold difference at a 98% confidence level. Similarly, we observed 78 genes with four-fold difference at a 95% confidence level and 35 genes with eight-fold difference between the hemmule and the blood vessel. These facts clearly indicate a very different nature of the hemmule and the bone marrow, lymph node, and blood vessel samples.

In the hemmule, we found three genes with 100 times larger RPKM values in comparison to those in the bone marrow. These genes include: Aif1l (allograft inflammatory factor 1-like), Gja4 (Gap junction alpha-4 protein), and Naa11 (N-alpha-acetyltransferase 11).

According to this analysis, hemmule has a very different gene transcript expression and representative gene distribution in comparison to the lymph node, BM, and blood vessel. This implies that the hemmule is different from the other three samples by a genetic nature. Further studies should help elucidate the reason and nature of this unique genetic profile.

### 2.3. Cells Found in the Hemmule

#### 2.3.1. Stem Cells and Gene Presentations in Hemmule by Immunoblotting and RNA-Seq

A stem cell niche is expected to have a large variety of cell including hematopoietic cells, mesenchymal stem cells, and endothelial cells [26]. In order to characterize hemmules, we analyzed gene expression for the selected niche, hematopoietic progenitor, mesenchymal, and osteogenic genes and antibodies to 16 cell type markers (Materials and Methods). Among them, RECA-1 and LYVE-1 were endothelial cell markers of blood and lymphatic vasculature, respectively [27]. The remaining 14 (Actin, Smooth muscle alpha-actin, CD146, CD90, CD133, CD150, Collagen-1, Fibronectin, NANOG, OCT4, REXO1, SOX2, SSEA-1, vWF) were stem cell markers commonly used in stem cell niche research and included mesenchymal, endothelial, and progenitor stem cell markers [15,28,29,30]. We did not test hemmule for the presence of pericytes.

The Correlation Matrix generated by PCA (S2 Table S1) shows that the variables, BV, BM, LN, and H are highly correlated. Many values are greater than 0.3. The main component variables are defined as linear combinations of the original variables. The Extracted Eigenvectors and Correlation tables (S2 Tables S2 and S3) indicates that first two principal components represent 86.4% of all data. The scree plot (Figure S3) confirms that PC1 and PC2 would suffice to describe all RNA results. The loading and score PCA plots are shown in Figures S4 and S5, respectively.

Figure 5a shows the PCA biplot combining the loading and score plots. The loading plot shows that vectors of all four variables are well separated in the space of PC1 and PC2 components indicating that in framework of PCA model, hemmule is considerably different from bone marrow, blood vessel, and lymph node. Protein expression corresponding to all 16 antibodies was measured in hemmule using Western blot. We show examples of Western blot in Figures S6–S8. The levels of these proteins line up with the expression of corresponding genes (Figure 5b), while the normalized RNA reads (RPKM) correlate with the relative protein levels measured by Western blot Figure 5c). Notably, the remaining six antibodies do not belong to this correlation. This finding is congruent with the analysis of protein and mRNA correlations and concludes that the lack of all-inclusive correlation shows the innate complexity of genetic regulation [31,32].

We found anti-CD90 cells by Western Blots of the hemmule with the positive controls in blood vessel and small intestine (Figure S6). The anti-CD90 antibody shows a trusted marker of mesenchymal and hemopoietic stem cells found in blood vessels [33] and intestine [34]. In addition, we located transcripts of Thy1 in the blood vessel (S1 Table S1). Similarly, the Western blot of anti-NANOG binding is shown in the samples of the hemmule along with the positive controls of blood vessel and small intestine (Figure S8). We also observed NANOG RNA transcripts in the blood vessel samples (S1 Table S1). Our findings of Western blot and RNA experiments underscore the variety of stem cells present in the hemmule. These results partly support the Schofield criteria for a stem cell niche as a space where stem cells can be maintained and replicated.

#### 2.3.2. Layered Structure and Cells in Hemmule Vessels Revealed by Immunohistochemistry

In the initial examination of the hemmule vessels, we concluded that they have a layered structure and they do not belong to blood or lymph vasculatures. These properties became the signature features of hemmule. Therefore, it is important to analyze further the layered nature and cells of hemmule.

Actin is the most abundant protein in most eukaryotic cells [35]. Smooth muscle alpha actin deserves special attention due to the presence of one of the few genes whose expression is somewhat restricted to vascular smooth muscle cells [36]. Figure S9 illustrates fluorescence images of cells within the cross-sections of hemmule immunostained with anti-smooth muscle alpha-actin antibody.

None of the studied antibodies produces such strong underpinning of the hemmule vessels as the smooth muscle of an anti-alpha actin antibody. The silhouettes of vessel section are strongly underlined at lower magnifications (Figure S10). The smooth muscle cells were observed in blood vessels (positive control) using high-resolution images (Figure S10d).

The anti-collagen-1 antibody does not reveal staining with the smooth muscle cells of Layer 2, but it does display binding with the longitudinal fiber cells in the outmost Layer 1 (Figure S11). 

**Figure 6:**
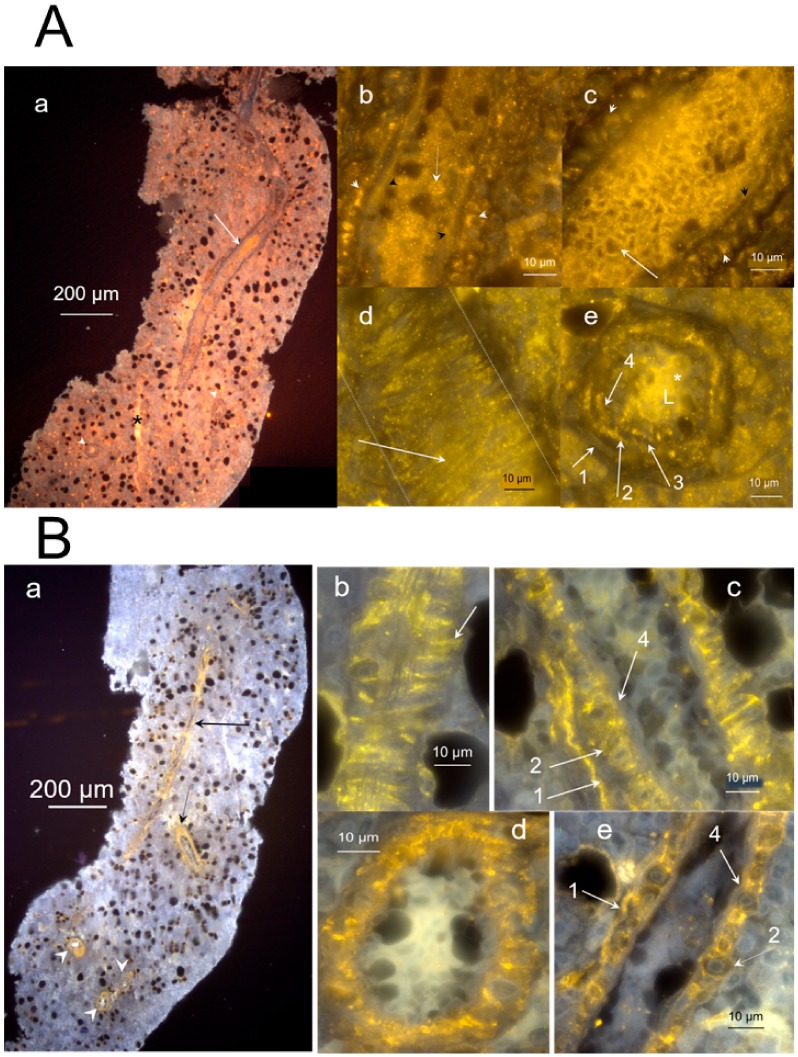
Layered structure of hemmule. (**A**) The sections of hemmule immunostained with the anti-CD150 antibody (**a**). The longitudinal cross-section. The inside portion of the large longitudinal vessel cells’ (arrow) lumen indicates positive staining involving anti-CD150 antibody. A small longitudinal channel is labeled by a black asterisk. Many stained cells are scattered in the entire area of the section. The lumens of transversal vessels are illustrated by white arrowheads. (**b**) The magnified fragment of the longitudinal vessel section. White arrowheads labels the cross-section of transversally positioned vascular smooth muscle of Layer 2. Black arrowheads marks the innermost Layer 4 of endothelial cells. The white arrow is shown to labels one of the cells inside the vessel of about 3 μm in diameter. (**c**) Another magnified fragment of the longitudinal vessel section. A white arrowhead is shown to label the cross-section of transversally positioned vascular smooth muscle cells of Layer 2. (**d**) Another fragment of the longitudinal vessel section. The area of the vessel between two dotted lines reveals transversal vascular smooth muscle cells (white arrow). (**e**) The transversal section of the vessel. Arrow 1 and 4 label the outmost and innermost layers of the vessel, respectively. The unstained Layer 3 is positioned between Layers 2 and 4. The cells inside the vessel are labeled by a white asterisk. L—Lumen. (**B**) The cross-section is immunostained with the anti-CD146 antibody. (**a**) The cells of longitudinal and transversal vessel walls (arrows and arrowheads, respectively) show binding of the antibody. (**b**). The magnified fragment of the longitudinal cross-section of the vessel is illustrated in the previou panel. The transverse thick vascular smooth muscle cells “ribs” is exemplified by one section and signified by the arrow. Many longitudinal fibers on the vessel’s long axis are visible in this fragment. (**c**). Another magnified latitudinal fragment of the section. Arrows 1 depict the layer of fibroblastic cells (Layer 1), whereas the layer of vascular smooth muscle cells (Layer 2) is illustrated by arrow 2. The layer of endothelial cells (Layer 4) is not stained (arrow 4). (**d**) The transversal section of the vessel. The vascular smooth muscle cells of Layer 2 are heavily stained. (**e**) Another magnified latitudinal fragment of the section. Arrows label Layers 1 and 2, respectively. Layer 4 is not stained (arrow 4).

The layered nature of the hemmule vessel wall is depicted by the interaction with anti-CD150 and anti-CD146 antibodies (Figure 6). The transversal section of the vessel (Figure 6A(e)) clearly reveals the presence of various layers in the vessel wall. The cross-section of transversally positioned layer of smooth muscle cells (Layer 2) is illustrated in Figure 6A panels b, d, e and panels b, d of Figure 6B. Panels b, c, e of Figure 6A clearly show Layers 1, 2, and 4.

Figure 6A(a) also reveals a difference between a hemmule vessel (arrow) and channel in the extracellular matrix (asterisk). The vessel does depict clear vessel walls, but the channel has no wall to separate the channel from the rest of the matrix. The interaction of anti-CD150 with Layer 2 of the smooth muscle cells seems unclear. We hypothesize that while anti-CD150 does not directly bind smooth muscle cells, it does bind the muscle-secreted glycoprotein [37]. Further discussion about the layered vessel structure will be undertaken in “diagram of a vessel inside a hemmule”.

#### 2.3.3. Individual Cells Stained by Stem Cell Antibodies in Hemmule

**Figure 7:**
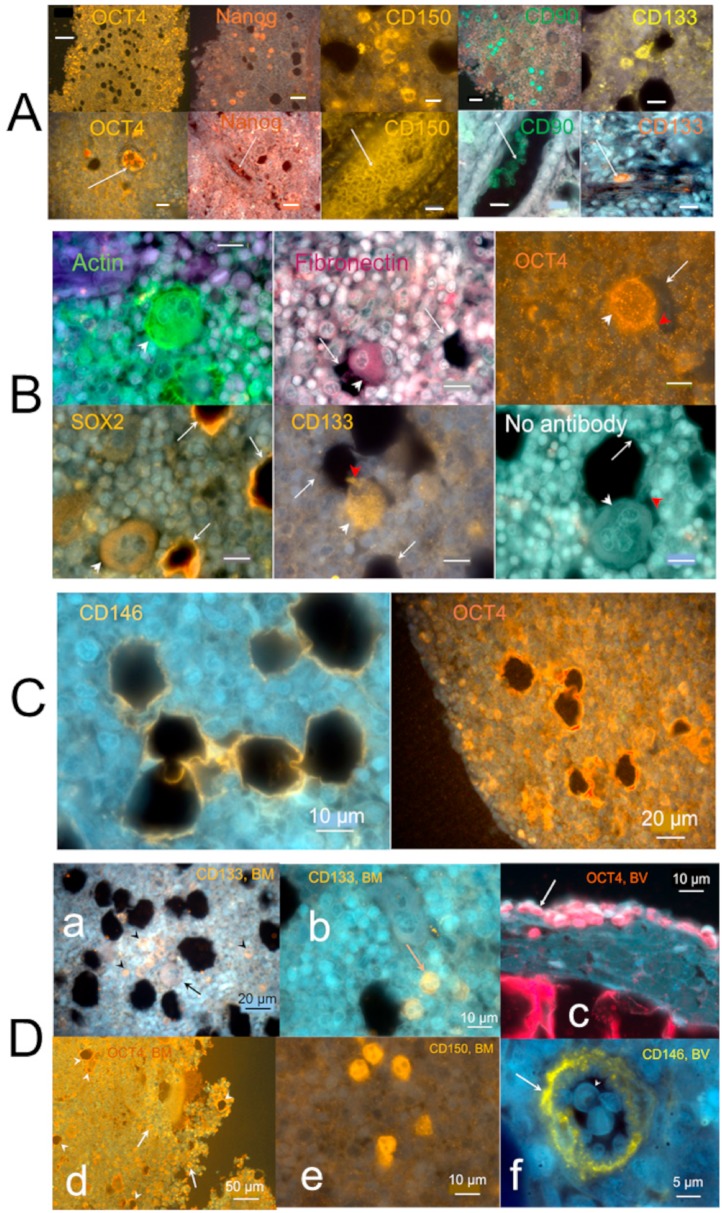
Individual hemmule cells stained by stem cell antibodies. (**A**) Fluorescent images of individual stem cell progenitors in the hemmule, which express embryonic cell markers. The top sections show individual stem cell progenitors being scattered in the hemmule cross-sections. The lower sections denote the progenitor cells inside the hemmule vessels L—lumen. Bars in the top panels, Oct4, 50 μm; Nanog, 20 μm; CD150, 10 μm; CD90, 20 μm; CD133, 10 μm. Bars in lower panels: Oct4, 20 μm; Nanog, 20 μm; CD150, 10 μm; CD90, 10 μm; CD133, 10 μm. (**B**) Large size cells in the hemmule belong to the megakaryocyte family. Some of these large sized cells illustrate small cytoplasmic extension (depicted by red arrowhead) and are in close proximity to the pores—the optically empty rounded regions (arrows). The formation of the most of these pores occurs when the large sized cells are displaced during the thin histological sample slicing. The rims of these pores may help retain molecules from these cells, which is evidenced by the staining by the antibodies. Bars: 10 μm. (**C**) Borders of the pores are stained by antibodies. (**D**) Both bone marrow and blood vessel cells stained by stem cell antibodies (positive controls). (**a**) Anti-CD133 positive cells in the bone marrow: the large size cell (arrow) and scattered cells (arrowheads). (**b**) Anti-CD133 positive scattered cells in bone marrow. (**c**) Anti-OCT4 antibody positive cells (arrow) on the surface of the blood vessel. (**d**) Anti-OCT4 antibody positive cells (arrows) and stained pores in bone marrow. (**e**) Anti-CD150 antibody cells in the bone marrow. (**f**) Anti-CD146 antibody stains the arteriole in the bone marrow (arrow).

In addition to the stem cells associated with BM vessels, the hemmule contains many progenitor stem cells scattered in the hemmule cross-sections and inside vessels (Figure 7A). The scattered cells interacting with anti-OCT4 antibodies are mostly distributed along the hemmule edges, while patches in the entire hemmule section (Figure 7A) distribute anti-Nanog, CD150, CD90, and CD133. The binding of cells with anti-CD150 antibody and the correlated relative value of RPKM of Slamf8 gene (Figure 5b) partially satisfies the requirements of SLAM code. According to the SLAM code demonstrated by Morrison and colleagues [38], the CD150^+^CD244^−^CD48^−^ cell population is highly enriched for hematopoietic stem cells.

The anti-antibodies of stem cells inside vessels revealed a different cell population with a maximal intra-vessel cell density inside the anti-CD150 antibody (Figure 7A). The distribution of OCT4 cells on the hemmule edges was estimated to be ten times higher in comparison to the remaining portion of the section (Figure 7A).

The presence of OCT-4 expression almost exclusively around the hemmule border assumes significance because the expression of OCT4 in bone marrow mesenchyme cells indicated osteogenic differentiation [39]. Similarly, the distribution of SOX2 scattered cells was predominantly found around the hemmule edges (Figure S12). This accumulation of SOX2 is significant because SOX2 is required for the osteoblasts’ self-renewal [40,41]. According to our finding, anti-OCT4 antibody stains large size cells, cells inside bone marrow vessels, cells around footprints, and are scattered around hemmule edges (Figure 7). In addition, we observed anti-SOX2 antibody cells in Layer 2, the footprints of cells, and scattered cells of different sizes (Figure 7).

Analyzing RNA-seq of bone marrow mesenchymal stem cells, Freeman and colleagues [42] found a group of genes associated with osteogenic differentiation. The group of osteogenic genes revealed by our RNA analysis in bone marrow is consistent with these findings. Although we did find the matching group of 29 osteopathic genes in bone marrow, we also found that the distributions of RPKM values of genes were different. S2 Table S4 depicts the RPKM levels of the osteogenic genes in BV, BM, LN, and H. Similarly, the gene Alpl (alkaline phosphatase) is present in the bone marrow at RPKM of only 3.6. The gene Alpl is paramount for osteoblast differentiation [43]. Therefore, the suggestion of osteoblasts being present on the border of hemmule appears to be credible. Notably, this suggestion is supported by the relatively high expression of CD146, which is also revealed by antibodies and RNA analysis of hemmules. CD146-positive osteoprogenitors defined a population of mesenchymal stem cells, which can produce osteoblasts [44,45]. These osteoblasts are believed to be one of the most critical components of stem cell niches [46]. The results of this section partly met the Schofield criteria, as per which stem cell niche is a site where stem cells could be maintained and replicate.

#### 2.3.4. Large Size Cells and Their Footprints

A few sections reveal larger size cells that are typically positioned in close proximity to the optically empty round areas (Figure 2d, Figure S13). Figure 7B (no antibody) shows a cell of about 25 μm in diameter located adjacent to the opening of the near identical size.

A few nucleoli are clearly visible inside the cell. This cell also reveals a small cytoplasmic extension (red arrowhead). Similar large size cells are immunostained by anti-actin, fibronectin, OCT4, SOX2, and CD133 antibodies (Figure 7B). The nucleoli are distinctly visible within the large cell in the anti-actin antibody panel of Figure 7B. Small cytoplasmic extensions (red arrowhead) are noticeable with anti-CD133 and OCT4 antibodies (Figure 7B). The anti-actin and OCT4 antibodies bind small immature hematopoietic cells enclosing the large size cells, while anti-fibronectin and anti-SOX2 are negative (Figure 7B). Some antibodies, such as Anti-SOX2 (Figure 7B), anti-CD146, and anti-OCT4 antibodies (Figure 7C), stain the edges of large cell footprints. Another image of the large size cell is illustrated in Figure S14.

Most of the pores are formed when the large sized cells are displaced during the thin histological sample slicing. It is our hypothesis that after being squeezed out during sample preparation, large size cells leave behind parts of their surface proteins that are then recognized by antibodies. The most likely candidate for the large size cells is the megakaryoblast family—megakaryoblast or promegakaryocytes [47]. Both conclusion and antibody interactions are consistent with the findings of extant literature on the immunohistochemistry of megakaryocytes. It has been demonstrated that the anti-actin antibody functions as a megakaryocyte marker in bone marrow samples [48]. It is also evident that stem cell differentiated into megakaryocytes correlates with Sox2 and Oct4 expression [49]. Similarly, CD133+ hematopoietic stem cells differentiated into megakaryocytic series [50] and megakaryocytes contribute to the bone marrow-matrix environment by expressing fibronectin [51]. As demonstrated by us, the large size cells are immunostained by anti-actin, fibronectin, OCT4, SOX2, and CD133 antibodies—consistent with the megakaryocyte nature of these large size cells. Based on only size and structure, the large size cell described in our research fits with the description of hemocytoblast [52,53,54]. The hemocytoblast in bone marrow is a primary stem cell of relatively large size (25–30 µm), round or oval nucleus, which takes almost the entire volume of the cell and leaves behind little space for cytoplasm. The cell reveals several very apparent nucleoli as well. The potential candidates for large size cells may also include giant macrophages or osteoclasts.

Panel (D) of Figure 7 illustrates positive control cells for the binding of anti-CD133, OCT4, CD150 antibodies inside the bone marrow and the blood vessel.

The presence of cells of megakaryocyte family assumes significance for homeostatic quiescence in stem cell niche. Therefore, it meets the Schofield requirement to have a place where cell differentiation was inhibited.

#### 2.3.5. Protein Allocation in Hemmule and Vessels Revealed by Immunohistochemistry and RNA-Seq Analysis

Immunohistochemistry showed that hemmule is composed of endothelial, hemopoietic, and mesenchymal cells, as depicted in Table 1.

The stem cells of hemmule identified by markers are allocated in vessel walls and inside the vessels; these cells are also scattered in the extracellular matrix/matrix channels. The hemmule contains mesenchymal, hemopoietic, and endothelial cells that are present in the stem cell niche [38,55]. The mesenchymal cells labeled by CD146, OCT4, SOX2, and CD90 can be seen in the hemmule vessel wall and inside the vessel. Additionally, the anti-CD90 antibody targets hematopoietic stem cells inside vessels and scattered in matrix. The anti-CD90 antibody is rarely positive in case of smooth muscle cells; however, DAPI stains cell nuclei and highlights the muscle cells along with anti-CD antibody (Figure S15).

Similarly, the hematopoietic stem cells were found to bind CD150 to cells inside vessels and scattered cells. The presence of embryonic stem cell-associated markers SSEA-1, Oct4, Nanog, and SOX2 was also identified in the hemmule. These findings are consistent with literature results specific to the mesenchymal and hemopoietic cell in BM. Research has shown that adult mesenchymal stem cell populations are derived from human bone marrow express embryonic stem cell markers OCT4, Nanog, alkaline phosphatase, and SSEA-4 [30]. The molecular signature of adult bone marrow-purified very small embryonic-like stem cells includes expression of Oct4, Nanog, SOX2, and SSEA-1 [56,57]. It appears as though the endothelial cells lining the internal surface of the hemmule vessel were not stained by any of the antibodies utilized in this work. However, cells inside the vessel, free scattered cells, and large size cells were found to bind with anti-CD133 antibodies. The CD133 is known to bind bone marrow endothelial cells [58,59]. According to Boltin et al. [58], circulating bone marrow–derived endothelial progenitor cells were positive for CD34, CD133, and anti-vascular endothelial growth factor (VEGF) receptor-2. On the other hand, CD34 cells depicted highly functional endothelial progenitor cells in the murine bone marrow [60]. Notably, our RNA-Seq analysis of MB node demonstrated relatively high reads (RPKM) for genes CD34, CD133, and Kdr (VEGF receptor-2) equivalent to 0.632, 0.687, and 2.963, respectively.

High-resolution fluorescent microscopy reveals that layered structure of BM vessels comprised of four layers (Table 1). The outermost Layer 1 of the vessel expressed the proteins labeled by anti- Fibronectin, Collagen-1, and NANOG, which assume importance for the environment of the stem cell niche [61]. Layers 1 and 2 are visible in many darkfield and fluorescent images. Layer 2 express mesenchymal cells shown by Oct4, Nanog, SOX2 (31), CD146 [44], and CD90 [62]. Layer 2 also strongly expresses smooth muscle α- actin and actin-binding cells, indicating the presence of smooth muscle cells. This finding is congruent with the report, according to which bone marrow mesenchymal cells differentiate after a vascular smooth muscle [63]. The feasible availability of smooth muscle cells in hemmule vessel is reinforced by detecting other proteins specific to smooth muscles. Our RNA-Seq analysis of genes found in hemmule revealed the presence of caldesmon, smooth muscle myosin heavy chains, calponin, actins, actin-binding proteins, gelsolin, as well as the cytoskeletal and extracellular matrix proteins, namely, collagen IV and elastin (Table 2).

The majority of relative numbers of reads (RPKM) pertaining to these proteins in the hemmule is comparable with those of bone marrow. However, it is notable that RPKM of the smooth muscle protein gamma 2 actin, and actin binding protein, kaptin, in the hemmule, are five and 10 times larger, respectively, than those of bone marrow (Table 2).

Layer 3 (Table 1) comprises of diffuse longitudinal muscle-like cells that are only visualized by Western blot with anti-Actin antibody. Layer 3 is depicted in Figure 3d, Figure 4i and Figure 6A(e). On the other hand, Layer 4 represents the innermost layer consisting of endothelial cells and is illustrated in Figure 3, Figure 4i and Figure 6A(e).

The endothelial cells are not stained in the remainder of the images. The presence of molecules indicative for endothelial cells in hemmule lends considerable support in favor of the endothelial nature of cells in Layer 4. The RNA-Seq analysis of hemmule revealed Esam (RPKM = 3.18), which indicates that the endothelial cell adhesion molecule and gene Esm1 (RPKM = 24.1) are responsible for endothelial cell-specific molecule. Importantly, it is evident that endothelial cells are not stained by the anti-LYVE-1 antibody (Figure 4i). The anti-LYVE-1 antibody stains intravascular cells, which reflects a high concentration of hyaluronan. The concentration of hyaluronan in the hemmule vessel can be estimated from the concentration of hyaluronan in rat BM [69] and those found by Western blot. According to our finding, hemmule contains hyaluronan in the range of 20–42 μg/g. The nature of endothelial cells belonging to Layer 4 of the hemmule layered structure is determined by the absence of interactions with anti-RECA-1 and anti_LYVE-1 antibodies. This, in turn, indicates that these endothelial cells do not belong to either the blood or lymphatic vasculatures. Meanwhile Table 1 shows that the bone marrow endothelial cells are positive with anti-actin and are also often positive with CD146 and Collagen-1 antibodies. The antibodies of scattered individual cells, large size cells and their footprint in cross-section of the hemmule, as well as cells inside the BM vessel are shown in Table 1 and described in the aforementioned sections “individual cells stained by stem cell antibodies in hemmule” and “large cells and their footprints”. The results of this paragraph, which include a large repertory of stem cells and their progenitor found in hemmule, support the Schofield requirement for a space where stem cells can be maintained and replicated.

#### 2.3.6. Genes Related to Functional Regulation of Stem Cells in the Hemmule Found by RNA-Seq Analysis

In order to determine the pattern of gene expression analyzed through our S1 Table S1, several genes of stem cell regulation were significantly expressed by the hemmule at the RPKM values as compared to those of the bone marrow (Table 3).

The vascular endothelial growth factor (Vegf) is involved in the presentation of Notch1 receptor; this receptor is required to maintain the quiescence and self-renewal of hemopoietic stem cells [70]. An extensive body of previous literature reveals that the effects of MYC and TIE2 on hemopoietic stem cells and N-cadherin expression appertain to a key role for N-cadherin in the retention of hemopoietic stem cells within the stem cell niche [71,72,73,74] indicating an important function of the niche [75]. The chemokine (C-X-C motif) receptor 4 and ligand of the tyrosine-kinase receptor encoded by Cxcl12 and Scf genes, respectively, participate in the maintenance of hematopoietic stem cells [76,77]. Meanwhile, research has shown that the expression glial fibrillary acidic protein activates transforming growth factor-β1, which, in turn, promulgates hematopoietic stem cells quiescence [78]. This quiescence has been regulated by megakaryocytes through the secretion of CXCL4 [79]. It is suggested that N-cadherin, TIE2, MYC, p21 and β-catenin pathways are interconnected, thereby hinting at the notion that they jointly control quiescence, self-renewal, and initiation of hemopoietic stem cell differentiation through their interaction with the niche [72,75]. The results show the presence in hemmule proteins that control quiescence, self-renewal, and initiation of hemopoietic stem cell differentiation. This meets the Schofield requirement to have a place after the inhibition of differentiation.

#### 2.3.7. Diagram of a Vessel Inside a Hemmule

**Figure 8:**
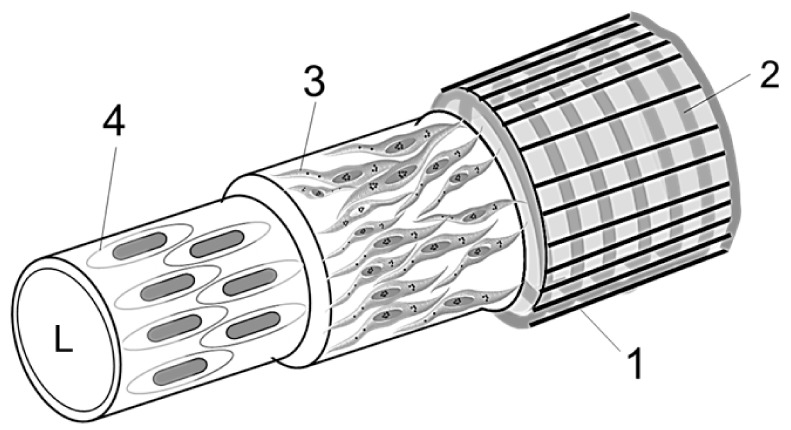
Diagram idealization of a vessel inside a hemmule. It comprises of four Layers: 1—the outermost layer of thin longitudinal fibers; 2—the adjacent layer is a continuous fibrous helix, or circumferentially oriented spiral that is composed of transversally positioned cells; 3—this layer comprises of thinly dispersed longitudinally oriented muscle-like cells; and 4—the innermost layer consisting of endothelial cells. L—Lumen.

Based on results obtained after conducting high-resolution microscopy, immunohistochemistry, and RNA-Seq analysis, we propose an idealized diagram of hemmule vessel structure. (Figure 8).

Layer 1 (Figure 8), the outmost layer of the hemmule vessel, mainly comprises of fibroblastic cells oriented on the main axis of the vessel and associated extracellular matrix surrounding the outer surface of adjacent Layer 2. Some images revealed collagen fibers with circumferential orientation Figure S11. The thickness of Layer 1 is 3–5 μm. The fibroblastic cells and fibroblast processes surrounding a vessel are clearly visible in Figure 3. The collagen 1 is clearly expressed in this layer.

Layer 2 (Figure 8), one of two middle layers of the hemmule vessel, is visible in almost all the sections and appears as a prominent structure. It mainly comprises of vascular smooth muscle cells. These cells average 4.6 μm in diameter and can reach 22–25 μm in length. The cells are arranged circumferentially around the vessel just underneath the outmost Layer 1, constituting a continuous helix. This helix has the following specifications: average diameter, D = 37.5 μm, cord diameter, d = 4.6 μm, pitch, p = 5.5 μm, and pitch angle, α = tan^−1^(p/πD) = 2.7°. The coil of vascular smooth muscle cells is reinforced by several cytoskeletal and extracellular matrix proteins, including collagen IV and elastin (detected by RNA-Seq).

Layer 3 (Figure 8) is another middle layer that primarily contains vascular smooth muscle cells. This layer comprises of longitudinally oriented thinly dispersed smooth muscle cells and bundles of collagen fibrils. Notably, this arrangement is very different from the circumferentially oriented tight helix alignment of muscle cells in Layer 2. The vascular smooth muscle cells are clearly visible in Figure 3. The average diameter of these muscle cells is 3.5 μm, whereas their length is up to 15 μm. The longitudinal bundle of fibrils belonging to Layer 3 is visible in Figure 3d and Figure 4i and Figure S2.

The Layer 4 (Figure 8) represents a continuous endothelium. On average, the endothelial cells are 5.7 μm wide and 18.6 μm long and oriented parallel to the long axis of the vessel. In this vessel, the endothelial cells are not stained by LYVE-1 antibody. The endothelial cell in Layer 4 passes together with a thin bundle of fiber, as illustrated in Figure 3c, Figure 4i and Figure 6B(c).

The four-layer structure of hemmule vessel depicts important information about the layered organization of arterial wall [80]. The arterial wall is composed of three major layers—the adventia, the outmost layer consists of fibroblasts and fibrocytes (similar to Layer 1 of hemmule vessel), the media, the middle layer, 3D network of smooth muscles, and fibers (similar to Layer 2 of the hemmule vessel), as well as intima, the innermost layer of endothelial cells (similar to Layer 4 of the hemmule vessel). In addition, a minor subendothelial layer is dominated by collagen with thinly dispersed smooth muscle cells (similar to Layer 3 of the hemmule vessel). The most prominent layer of hemmule vessel (Layer 2), the layer vascular smooth muscle cells comprises of helix where cells arranged circumferentially and coherently around the vessel are very similar to the muscle helix of the aorta wall. The significant difference between the aorta and hemmule vessel walls lies in the diameter of the vessel. The aorta diameter varies between 1–3 cm [81], whereas the hemmule vessel size is between 10–50 µm. Additionally, aorta carries blood cells, but the hemmule contains stem cells and their progenitors. The general structure of the hemmule reveals a fair comparison with the layered structure of arterioles. The electron microscopic studies of cat mesenteric arterioles [82] revealed three major structural layers—intima, media, and adventitia—although they appeared quite irregular. The external fibrils were oriented longitudinally, while the vascular smooth muscles were positioned circumferentially and helically, similar to the hemmule vessel cells in Layers 1 and 2, respectively. Unlike the regular and smooth lumen of the hemmule vessel, the vessel lumen of the arterioles was found to be irregular. The subendothelial layer contained fibers and did not exhibit longitudinally oriented smooth muscle cells, as observed in Layer 3 of the hemmule vessel. The average diameter of arteriole was ~30 μm, whereas the lumen of arteriole was filled up by blood cells. Although some similarities do exist between the structural organization of arteries, arterioles, and hemmule vessels, their differences are significant.

The vessels are critical components of a 3D hemmule model. The 3D model of the hemmule is in progress.

## 3. Discussion

### Hemmule as a Stem Cell Niche

Schofield [22] proposed the concept of stem cell niche, where specific niche cells establish close interactions with immature cells that can subsequently enforce stem cell behaviors, including proliferation and inhibition. Some structural features and cell composition of the hemmule are comparable with that proposed for stem cell niches.

Therefore, the first Schofield requirement for the stem cell niche to entail a distinct anatomical position is met. Our finding suggests that the hemmule is positioned in the bone diaphysis. It can be easily located and lifted, as described in our experimental section.

The second Schofield requirement is to present a site where stem cells can be maintained and can replicate. The cellular composition of the hemmule is consistent with this requirement. The osteoblasts are concentrated within the periphery, embracing the entire hemmule. In this regard, we propose that the thin capsule enclosing the hemmule and peripheral osteoblasts can provide a protective microenvironment that facilitates the maintenance and self-renewal of stem cells by safeguarding them from uncontrolled differentiation and apoptosis. In this regard, our hypothesis is that CD146-positive progenitors, in conjunction with the hemmule vascular system, give rise to osteoblasts and organize a hemopoietic niche, which is similar to how osteoblasts produce a hemopoietic niche after xenotransplantation along with the sinusoidal endothelial cell [83]. Hematopoietic stem cells are known to produce all blood cells. The equilibrium between stem cell proliferation and quiescence is controlled to safeguard blood homeostasis whilst simultaneously lowering the extent of cellular damage because it is important for stem cells to endure for a lifetime. As a result, cell cycle regulation assumes great significance when it comes to guiding stem cells [84].

The third Schofield requirement is that the stem cell niche is where differentiation is inhibited. Consistently with this requirement, we have found evidence in our experiments. The genes related to proteins (N-cadherin, TIE2, MYC, p21 and β-catenin) controlling quiescence, self-renewal, and initiation of hemopoietic stem cell differentiation were well presented in hemmule by RNA analysis. The hemmule has large sized cells with a structural appearance and antibody profile that fits into megakaryocytes, which regulate and maintain homeostatic quiescence whilst also contributing to the environment of bone marrow matrix [51,79,85]. However, our data (S1 Table S1) show that genes IL7 and CSF1 are overexpressed in hemmules (Il7: BV, 1.246; BM, 0.377; LN, 1.668 and Hemmules, 3.481; Csf1: BV, 4.191; BM, 6.934; LN, 0.939 and Hemmules, 5.674). Recent data indicate that these genes are involved in differentiation of lymphoid and myeloid cells, respectively [86,87,88]. Considering these facts, the third Schofield requirement needs to be further investigated.

Finally, the fourth Schofield requirement is to have a restricted space that limits the number of stem cells. We suggest that the limited size of hemmule controls stem cell proliferation due to the close proximity of cells to each other within the confined space. Indeed, the proliferation rate of hematopoietic stem cells is restricted by the size of extracellular matrix functionalized microcavities [89].

Moreover, the spongy extracellular matrix of the hemmule containing channels and vessels provide a convenient microenvironment for intercellular communication and cell transport. The hemmule reveals the presence of large repertory of mesenchymal, hemopoietic, and endothelial stem cells. In turn, this confirms that the hemmule is a suitable candidate for the combined stromal and vascular stem cell niche. In future experiments we will isolate hemmules from bone marrow, digest them, culture the cells, and immunostaine them for the presence of stem cells markers, which will further prove if hemmule structure is niche for stem cells, and which types of stem cells are there.

Overall, our results suggest that hemmules are the hematopoietic niches and offer methods and approaches required to test this conclusion further. Hemmules are easy to harvest and study *ex-vivo* in varying environments to evaluate transition from rat to human models, study oncogenesis mechanism of cell activation and inhibition, as well as other processes of cell interactions and communication. If our conclusion is correct, studies of hemmules in animals and humans may provide yet to be explored approaches to regenerative medicine and other areas of research and medicine where stem cells can be used for therapeutic need.

## 4. Materials and Methods

### 4.1. Animals

The animal protocol was approved by the Auburn University Institutional Animal Care and Use Committee (AU IACUC) (ethical protocol code 2016–2927, 14 November 2018). Adult male Sprague-Dawley rats (Envigo, Dublin, VA, USA) weighing ~300 g were used.

### 4.2. Microdissection and Extraction of Hemmules

A femur bone was split into two halves using a scalpel and making small, closely spaced holes longitudinally along the two sides of the bone. The opening of these two halves exposed the bone marrow (BM). Exploratory movements by surgical tweezers showed vessels with hemmules that are not attached to the BM matrix in the bone diaphysis and can be lifted. The hemmules were removed using surgical scissors and fixed in Bouin’s fluid (Electron Microscopy Sciences, Hatfield, PA, USA). We successfully collected 4–12 hemmules from one bone. Simultaneously, we also collected control samples of BM blood vessels, BM, and lymph nodes. The findings presented in this work represent typical samples obtained from a total of 42 rats, 190 hemmules, and 1200 sections. Some sections were sliced further by means of optical slicing in order to view sections underneath the cutting surface.

### 4.3. Immunohistochemistry

Following the fixation in Bouin’s fluid, the hemmules were placed in cassettes and paraffin infiltrated in a Tissue Tek VIP processor (Rankin Biomedical Corporation, Oakland County, MI, USA). These tissues were embedded in paraffin, and 6 µm sections were mounted atop glass slides. The sections were then deparaffinized in Hemo-De (Scientific Safety Solvents, TX, USA). Subsequently, these sections were hydrated with an ethyl alcohol series of descending dilutions of 100, 95, 70, and 0% using distilled water. These sections were permeabilized in 0.1% TritonX-100 (Sigma-Aldrich, MO, USA) and humidified before being blocked with 5% goat or donkey serum at room temperature for one hour. Blocked sections were exposed to the following antibodies diluted in 5% goat or donkey serum in PBS: Actin (1:100, Millipore, Burlington, MA, USA; MAB1501), Smooth muscle alpha actin (1:50, ThermoFisher Scientific; PA5-18292), CD146 (1:100, abcam; ab75769), CD90 (1:100, ThermoFisher Scientific; MA1-80651), CD133 (1:20, ThermoFisher Scientific; 18470-1-AP), CD150 (1:50, ThermoFisher Scientific; PA5-21123), Collagen 1 (1:50, Novus Biologicals; ND600-408), Fibronectin (1:50, ThermoFisher Scientific; 15613-1-AP), LYVE-1 (1:100, ThermoFisher Scientific; PA1-16635), RECA-1, 1:100, abcam; ab9774), NANOG (1:100, ThermoFisher Scientific; PA5-20889), OCT4 (1:50, ThermoFisher Scientific; PA5-20887), REXO1 (1:20, ThermoFisher Scientific; 13503-1-AP), SOX2 (1:100, abcam; ab7959), SSEA-1 (1:100, abcam; ab16285), vWF (1:20, ThermoFisher Scientific; MA5-14029). These sections were thoroughly washed in Copling Jar for two hours prior to the application of secondary antibodies. Subsequently, the slides were incubated in the dark with secondary antibodies in blocking buffer (5% serum) at room temperature for one hour: Alexa Fluor 488 or Alexa Fluor 555 (1:500, ThermoFisher Scientific). Slides were subsequently washed in copling jar with PBS and 0.01% Tween-20, dehydrated, mounted with Eukitt mounting media (Sigma-Aldrich), and cover-slipped. Some slides were stained with Hematoxylin and Eosin (H&E). All slides were stored at a temperature of 4 °C in the dark.

### 4.4. Western Blots

Hemmules, along with samples of bone marrow, lymph node, and blood vessel, were extricated from each rat, snap-frozen in liquid nitrogen, and kept at −80 °C until use. Tissues were homogenized using T-PER reagent with protease inhibitor cocktail (Thermo Scientific, Rockford, IL, USA). Subsequently, samples were centrifuged at 15,000× *g* for 30 min at 4 °C, after which supernatants were gathered. A protein assay (Bio-Rad) was conducted in order to determine the protein concentration for each sample. Thereafter, an equal amount of proteins (50 µg) was separated by SDS–PAGE (10%) before being transferred into nitrocellulose membranes. These membranes were blocked for 1 h in Odyssey blocking buffer (LiCor, Lincoln, NE, USA) and incubated overnight at 4 °C with primary antibodies. The membranes were washed with PBS/0.1% Tween-20 three times, incubated with goat anti-rabbits IRDye 800CW secondary antibodies for 1 h, and then washed with PBS/0.1% Tween-20 four times. Membranes were imaged by LiCor Odyssey scanner, and blots were analyzed by Image Studio 2.0 analytical software (LiCor, Lincoln, NE, USA). The assay was repeated at least four times for each protein. Bands were calibrated by loading of samples of intestine (Figure S15) and were then normalized to the density of smooth muscle α-actin, represented as a ratio of each protein to α-actin. 

### 4.5. RNA Analysis

RNA-Seq analysis was conducted by MR DNA (Shallowater, TX, USA). Three samples of each hemmule, blood vessel, bone marrow, and lymph node were carefully excised, immediately transferred into PBS and washed with PBS. Each sample was processed individually. One ng-500 ng of total RNA was used to prepare the library. Total RNA was isolated from the samples using the RNeasy PowerLyzer Tissue&Cells Kit (Qiagen, Germantown, MD, USA) in accordance with the manufacturer’s instructions. The concentration and purity of total RNA was determined using the Qubit RNA Assay Kit (Life Technologies, Waltham, MA, USA) prior to cDNA conversion. One ng-500 ng of total RNA was used to prepare the library using the TruSeq RNA LT Sample Preparation Kit (Illumina, San Diego, CA, USA) by following the manufacturer’s instructions. Following the library preparation, the final concentration of the libraries was measured using the Qubit dsDNA HS Assay Kit (Life Technologies, Waltham, MA, USA). Similarly, the average library size was determined using the Agilent 2100 Bioanalyses (Agilent Technologies, Santa Clara, CA, USA). The final RNA concentration of these libraries was at average of 11 ng/μL, and the average library size was 404 bp. These libraries were pooled, diluted (to 8.0 pM) and sequenced paired end for 300 cycles using the HiSeq system (Illumina San Diego. CA, USA). Additionally, DNASTAR’s Genomics Suite was used to complete the alignment of RNA-Seq the *Rattus norvegicus* genome (*Rattus_norvegicus-Rnor*_6.0). The data file of all three samples contains 519 genes (S1 Table S1). Thereafter, gene expression comparisons were made between the hemmule (H) and the three remaining groups of interest: Blood Vessel (H-vs.-BV), Bone Marrow (H-vs.-BM), and Lymph Node (H-vs.-LN). It is notable that these comparisons were completed at 95% and 98% confidence intervals, respectively. Comparisons were then concluded using a 4-fold and 8-fold change thresholds, respectively between the groups. A total of six gene comparison sets were calculated: H vs. BV, (95%) 78 genes, H-vs.-BM, (95%) 17 genes, H vs.-LN, (95%) 147 genes, H vs. BV, (98%) 35 genes, H-vs.-BM, (98%) 6 genes, and H vs.-LN, (98%) 32 genes. For each gene, the number of reads assigned per kilobase per million mapped reads (RPKM) was measured in all six comparison sets and used as an indicator of sample comparison.

### 4.6. High-Resolution Fluorescent Microscopy

The fluorescent optical system comprised of a specially-designed condenser [90] positioned in an Olympus BX51 microscope (Olympus America Inc., Center Valley, PA, USA) by replacing a bright-field condenser. Light from a light source (EXFO120, Photonic Solution Inc., Ont., Canada) was connected to the condenser using a liquid light guide through the dual mode fluorescent filter [91]. These images were observed by utilizing Olympus 100X UPlanApo Oil Iris objective and fluorescence quad filters, Chroma 89101x and 89101m (Chroma Technology Corp., Bellows Falls, VT, USA) and recorded with Zeiss AxioCam camera (Carl Zeiss Inc., Thornwood, NY, USA); 90 nm resolution in these images was achieved using an optical illumination system with a high-aperture cardioid annular condenser [90]. The optical system offers the ability to produce optical sectioning. Meanwhile the optical sectioning also permits the discerning of in-focus image from out-of-focus structures. Additionally, it allows the user to see not only the sample-cutting surface, but also get a glance at the inside portion of optical sections below the cutting plane. Fine focusing and placement of focus in any depth of the sample allows the focus to be placed at a desirable increment. Accordingly, a three-dimensional profile of the sample can be obtained. Furthermore, the dual mode optical system allows simultaneous recording of fluorescent and high-resolution darkfield images [91].

### 4.7. Statistical Analysis

Principal component analysis, Data averaging, ANOVA, curve fitting, and graph plotting were conducted using Origin 2019 (Northampton, MA, USA) and 2010 Microsoft Excel. RNA-seq data comparisons between the hemmule and blood vessel (H-vs.-BV), bone marrow (H-vs.-BM), and lymph node (H-vs.-LN) were carried out using one-way ANOVA. This was then followed by Tukey’s multiple comparison test. Similarly, the RPKM values were compared for Habp4 (expressing hyaluronan binding protein) in the hemmule and blood vessel, bone marrow, and lymph node using one-way ANOVA. Additionally, the linear correlation analysis was used for lining up RPKM and relative levels of corresponding proteins.

### 4.8. Principal Component Analysis Plot

Principal component analysis (PCA) plot was generated using Origin 2019 package with RPKM values. The 37 selected niche genes, hematopoietic progenitor, mesenchymal, and osteogenic genes (Acta2, Mcam, Thy1, Prom1, Slamf8, Fn1, Nanog, Lyve1, Cd34, Habp4, Col18a1, Aif1l, Naa11, Gja4, Il7, Naa11, Cdh2, Alpl, atf4, Bglap, Bgn, Bmp4, Bmp6, Col1a1, Csf1, Dcn, Igfbp3, Il18, Jund, Kitl, Mmp13, Nfatc1, Ogn, Runx2, Sp7, Sparc, and Spp1) were used for analysis. Extracting four components, PCA analysis resulted in the Correlation Matrix, Eigenvalues of the Correlation Matrix, Extracted Eigenvectors, and Scree, Loading, Score, and Biplot plots.

## Figures and Tables

**Table 1:** Distribution of cell markers in hemmule and vessels.

Antibody	Layer 1 ^1^	Layer 2 ^2^	Layer 3 ^3^	Layer 4 ^4^	Inside ^5^	Large Cells ^6^	Footprints ^7^	Scattered ^8^
α-Actin	−	+	−	−	−	−	−	−
Actin	−	+	+	+	+	+	−	+
CD146	−	+	−	+−	+	−+	−+	−
CD90	−	−+	−	−	+	−	−	+
CD133	−	−	−	−	+	+	−	+
CD150	−	+	−	+−	+	−	−	+
Collagen-1	+	−	−	+−	+	−	−	−
Fibronectin	+	+	−	−	−	+	−	−
LYVE-1	−	−	−	−	+	−	−	−
Nanog	+	+	−	−	+	−	−	+
OCT4	−	−	−	−	+	+	+	+
RECA-1	−	−	−	−	−	−	−	−
REXO1	−	−	−	−	−	−	−	+
SOX2	−+	+	−	+−	−	+−	+	+
SSEA-1	−	−	−	−	−	−	−	+
vWF	−	+	−	−	+	−	−	+

^1^ The outermost layer of the vessel mainly consists of fibroblasts and fibrocytes, collagen and fibrinogen fibers organized in longitudinal bundles. ^2^ One of the middle layers comprises of three-dimensional network of transversally positioned smooth muscle cells. ^3^ Layer is composed of thinly dispersed longitudinally oriented muscle-like cells. ^4^ Innermost layer comprise of endothelial cells. ^5^ Cells inside BM vessels. ^6^ Large size cells in the hemmule. ^7^ The rims of pores that retain molecules from the large size cells. ^8^ Cells scattered in the hemmule cross-section. +—most cells positive, −—negative, +−—often positive, −+—rarely positive.

**Table 2:** Genes and functional proteins for vascular smooth muscle lineage.

Gene	H	BM	Protein	Ref.
	RPKM	RPKM		
Cald1	52.00	54.23	Caldesmon 1	[64]
Mybph	0.902	0.629	Myosin binding protein	[65]
Myh11	0.846	0.538	Myosin, heavy chain 11, smooth muscle	[66]
Acta2	15.61	10.842	Actin, alpha 2, smooth muscle, aorta	[67]
Actg2	0.409	0.083	Actin, gamma 2, smooth muscle, enteric	[68]
Cnn3	44.46	57.44	Calponin 3	[68]
Kptn	2.650	0.26	Kaptin (actin binding protein)	[68]
Gsn	15.650	29.215	Gelsolin	[68]
Eln	7.223	1.427	Elastin	[68]
Col4a1	7.223	4.451	Collagen type IV alpha 1 chain	[68]
Col4a2	3.880	2.974	Collagen type IV alpha 2 chain	[68]

Gene—the gene related to the proteins for vascular smooth muscle lineage; H—hemmule; BM—bone marrow; RPKM—Reads assigned Per Kilobase per Million mapped reads; Protein—functional proteins for vascular smooth muscle lineage; Ref.—NCBI references for key tissues for each gene.

**Table 3:** RPKM values of genes and proteins involved in stem cell regulation.

Gene	BV	BM	LN	H	Protein
Tek	1.90	3.50	0.46	2.42	TEK receptor tyrosine kinase, TIE2
Cdh2	1.14	0.10	0.14	1.06	N-cadherin
Kdr	3.11	2.90	1.04	2.96	Vegfr-2, vascular endothelial growth factor
Notch1	0.91	0.74	0.90	0.84	Notch signaling protein
Myc	3.69	7.14	21.6	3.93	Myelocytomatosis oncogene protein
Krcc1	22.8	67.6	21.6	28.4	P21—cyclin-dependent-kinase
Ctnnb1	50.8	79.8	9.90	56.4	β-catenin
Cxcl12	361	516	11.7	463	Chemokine (C-X-C motif) receptor 4
Scf	16.1	13.9	0.35	18.2	Ligand of the tyrosine-kinase receptor
Tgf-β1	29.3	62.1	32.5	36.7	Transforming growth factor beta-1
Gfap	0.72	1.1	0.13	0.75	Glial fibrillary acidic protein
Cxcl4	265	796	6.1	204	Platelet factor 4

BV—blood vessel; BM—bone marrow; LN—lymph node; H—hemmule; Protein—proteins involved in stem cell regulation.

## Supplementary Materials

Supplementary materials can be found at https://www.mdpi.com/1422-0067/21/2/539/s1.

## Abbreviations

ANOVA	Analysis of variance
BM	Bone marrow
BV	Blood vessel
H	Hemmule
LN	Lymph node
RPKM	Reads assigned per kilobase per million mapped reads
