# Supplementary material for: Hemmule: A Novel Structure with the Properties of the Stem Cell Niche
Source: Int J Mol Sci. 2020 Jan 14;21(2):539. doi: 10.3390/ijms21020539 (PMC7013657; doi:10.3390/ijms21020539)
Supplement: Supplementary file 1 [file ijms-21-00539-s001.zip › Supporting information files/S2 Supplementary materials.docx]

**S2 Supplementary materials**

Stem cell node.

Vitaly Vodyanoy,^1,2^ Oleg Pustovyy,^1^ Ludmila Globa,^1^ Randy J Kulesza Jr^3^ and Irina Sorokulova^1,2^

^1^Department Anatomy, Physiology and Pharmacology, College of Veterinary Medicine Auburn, AL 36849; ^2^School of Kinesiology, Auburn University, Auburn, AL 36849, ^3^Lake Erie College of Osteopathic Medicine, Erie, PA 16509

Correspondence should be addressed to Vitaly Vodyanoy, vodyavi@auburn.edu


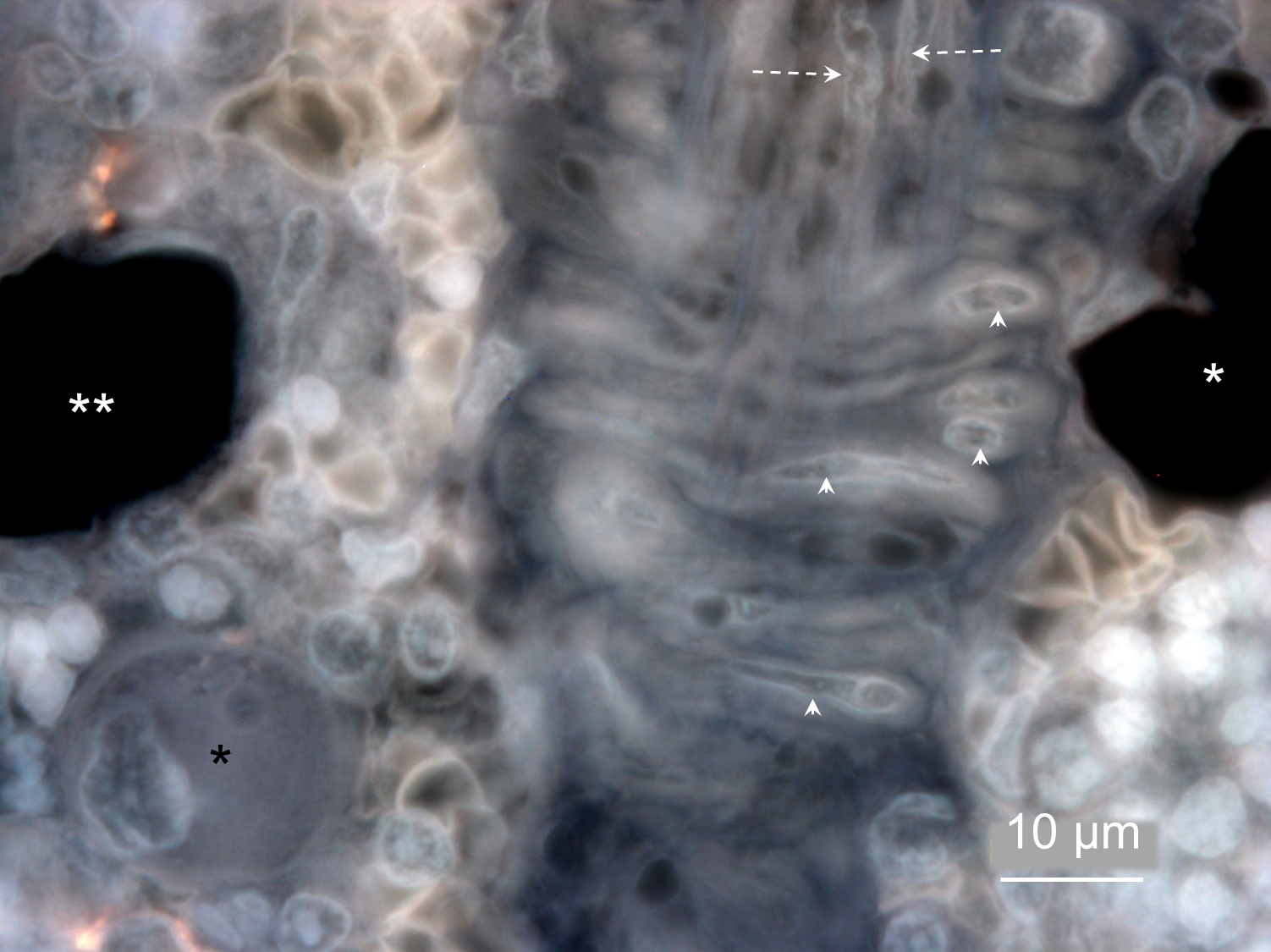


**Figure S1** High magnification images of the vessel inside hemmule. The color is produced by auto-fluorescence. a. Longitudinal section of the vessel. The characteristic features of this section are vining around the vessel thick fibers loops. The cell constituting these fiber loops are labeled by arrowheads. Transversal cells belonging to another vessel layer are labeled by dotted arrows. The footprint of a large cell is labeled by white asterisk, and the orifice of the small transversal vessel is identified by two white asterisks. The large round cell is marked by black asterisk.


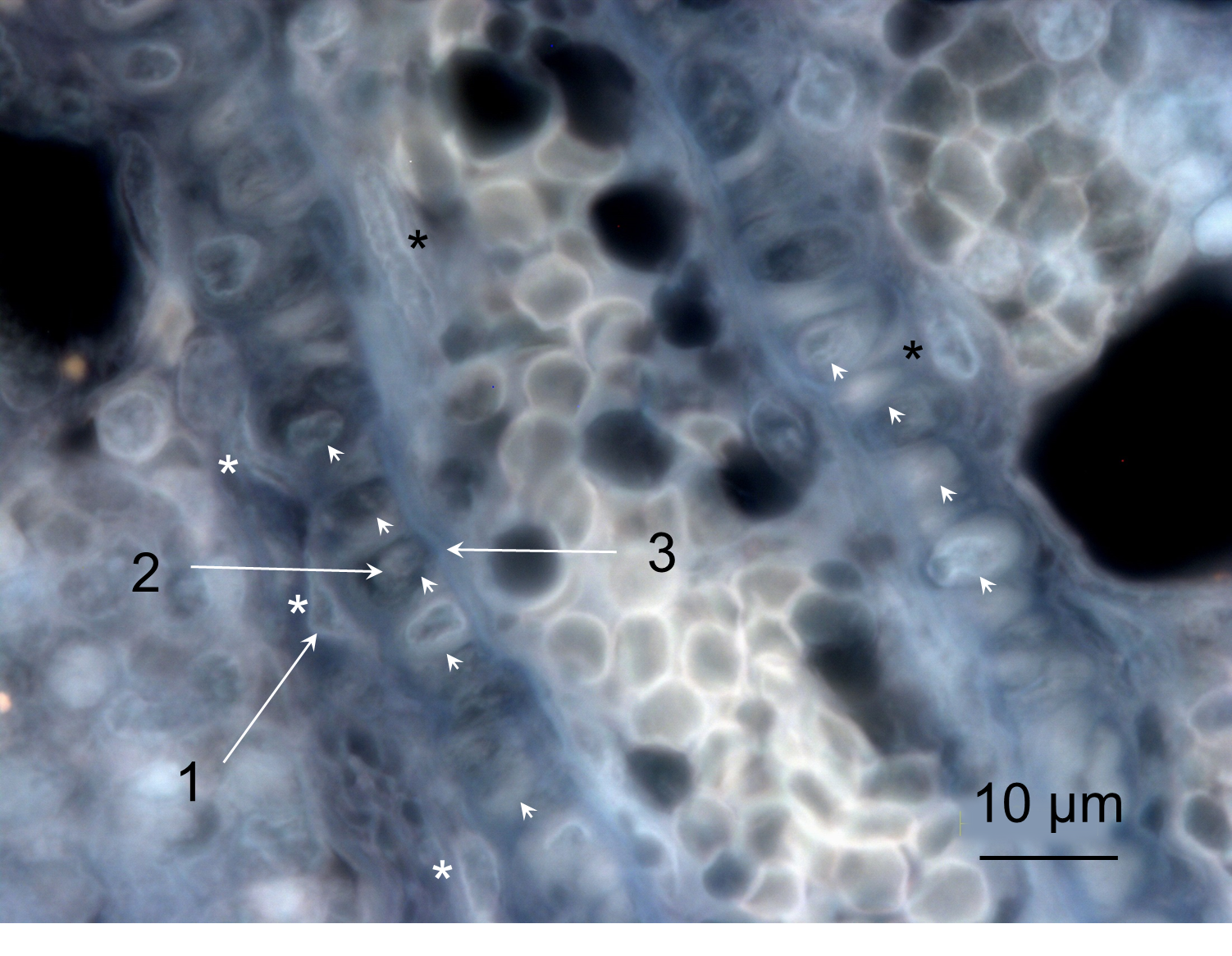


**Figure S2.** The longitudinal section cutting the middle of the vessel. Pseudocolor is due to autofluorescence. Layers 1-3 are labeled by white arrows. The external layer 1 is represented by cells positioned along the layer (white asterisk). Layer 2, a thick transversal fiber waving around the vessel is shown by cross-section of the cells positioned perpendicular to the section plane on both sides of the vessel (white arrowhead). Layer 3 positions longitudinally and does not show cells in this image. The internal layer 4 is composed of longitudinal cells (black asterisks).

**Principal component analysis**

The principal component analysis has been applied to RPKM values of the 37 selected niche genes, hematopoietic progenitor, mesenchymal, and osteogenic genes (Acta2, Mcam, Thy1, Prom1, Slamf8, Fn1, Nanog, Lyve1, Cd34, Habp4, Col18a1, Aif1l, Naa11, Gja4, Il7, Naa11, Cdh2, Alpl, atf4, Bglap, Bgn, Bmp4, Bmp6, Col1a1, Csf1, Dcn, Igfbp3, Il18, Jund, Kitl, Mmp13, Nfatc1, Ogn, Runx2, Sp7, Sparc, and Spp1).

**S2 Table S1**. Correlation Matrix

|  | BV | BM | LN | H |
| --- | --- | --- | --- | --- |
| BV | 1 | 0.91693 | 0.17879 | 0.38723 |
| BM | 0.91693 | 1 | 0.36855 | 0.51879 |
| LN | 0.17879 | 0.36855 | 1 | 0.4787 |
| H | 0.38723 | 0.51879 | 0.4787 | 1 |

**S2 Table S2**. Eigenvalues of the Correlation Matrix

|  | Eigenvalue | Percentage of Variance | Cumulative |
| --- | --- | --- | --- |
| 1 | 2.47112 | 61.78% | 61.78% |
| 2 | 0.98377 | 24.59% | 86.37% |
| 3 | 0.48562 | 12.14% | 98.51% |
| 4 | 0.05949 | 1.49% | 100.00% |

**S2 Table S3**. Correlation coefficients

|  | Coefficients of PC1 | Coefficients of PC2 | Coefficients of PC3 | Coefficients of PC4 |
| --- | --- | --- | --- | --- |
| BV | 0.53884 | -0.50228 | 0.12912 | 0.66385 |
| BM | 0.59399 | -0.29505 | 0.14664 | -0.73391 |
| LN | 0.36753 | 0.71282 | 0.58358 | 0.1275 |
| H | 0.4709 | 0.39059 | -0.7882 | 0.06661 |

PC1= 0.53884×BV+0.59399×BM+0.36753×LN+0.4709×H

PC2= -0.50228×BV-0.29505×BM+0.71282×LN+0.39059×H

PC3= 0.12912×BV+0.14664×BM+0.58358×LN-0.7882×H

PC4= 0.66385×BV-0.73391×BM+0.1275×LN+0.06661×H

**Figure S3**. Scree Plot.

**Figure S4**. Loading Plot

**Figure S5**. Scores Plot


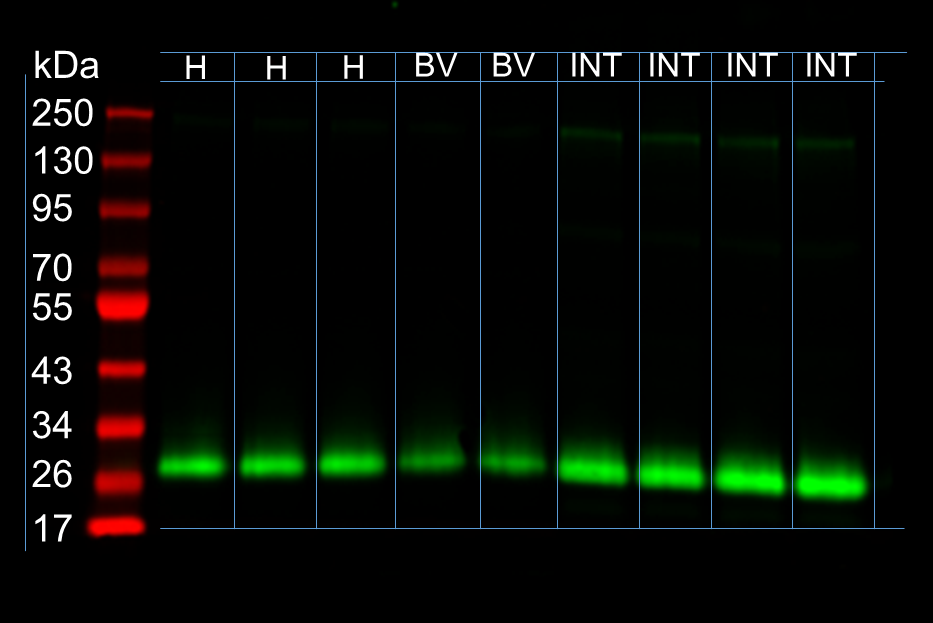


**Figure S2 6.** Western blot for the anti-CD90 antibody on the samples of the hemmule (H), blood vessel (BV), and small intestine (INT) (positive control).


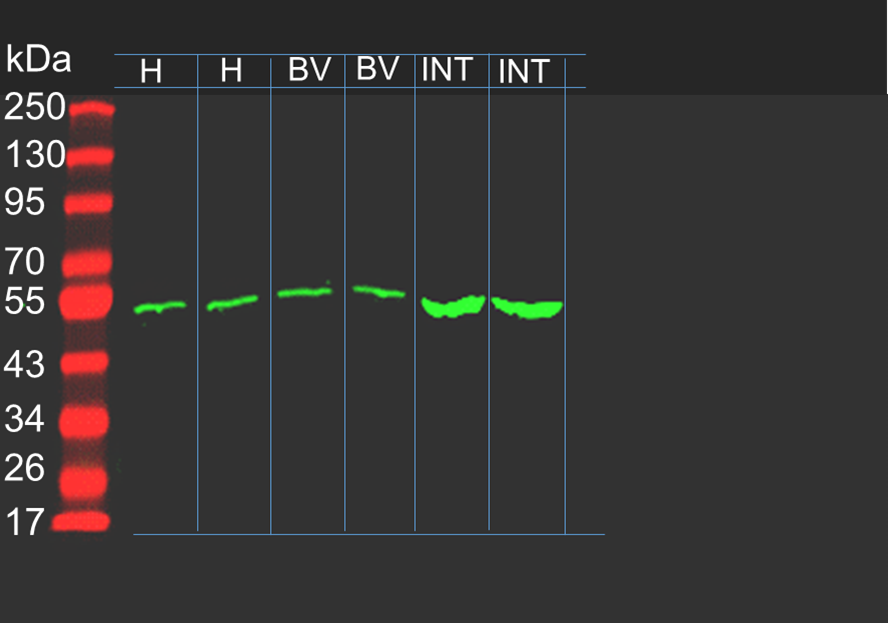


**Figure S7.** Western blot for the anti-SOX2 antibody on the samples of the hemmule (H), blood vessel (BV), and small intestine (INT) (positive control).


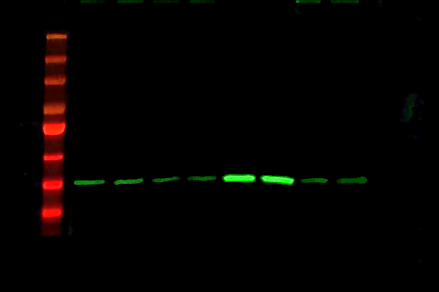


26

34

43

55

70

95

130

250

kDa

BMN

BMN

BV

BV

BM

BM

INT

INT


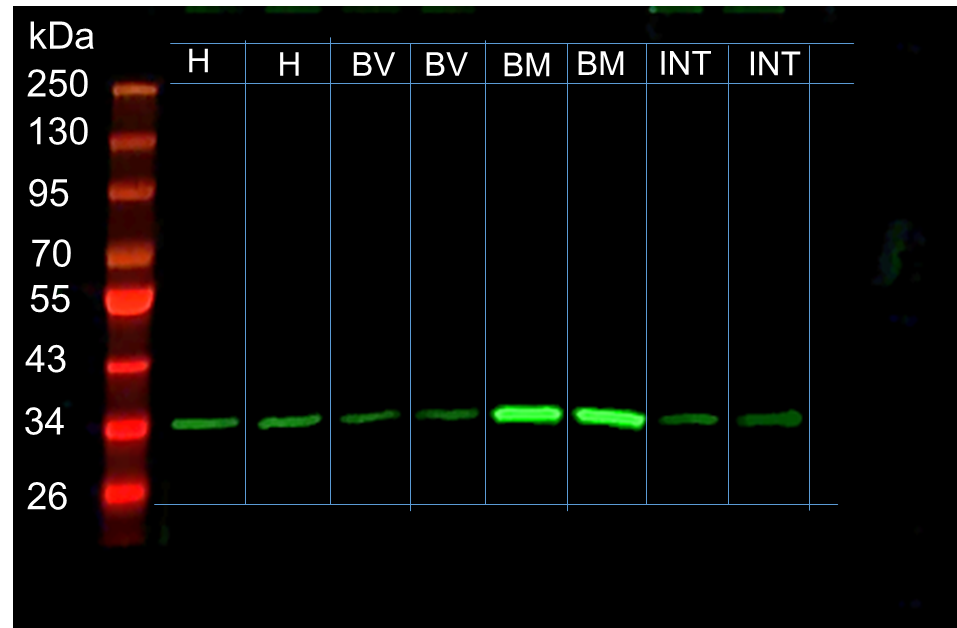


**Figure S8.** Western blot for the anti-NANOG antibody on the samples of the hemmule (H), blood vessel (BV), and small intestine (INT) (positive control).


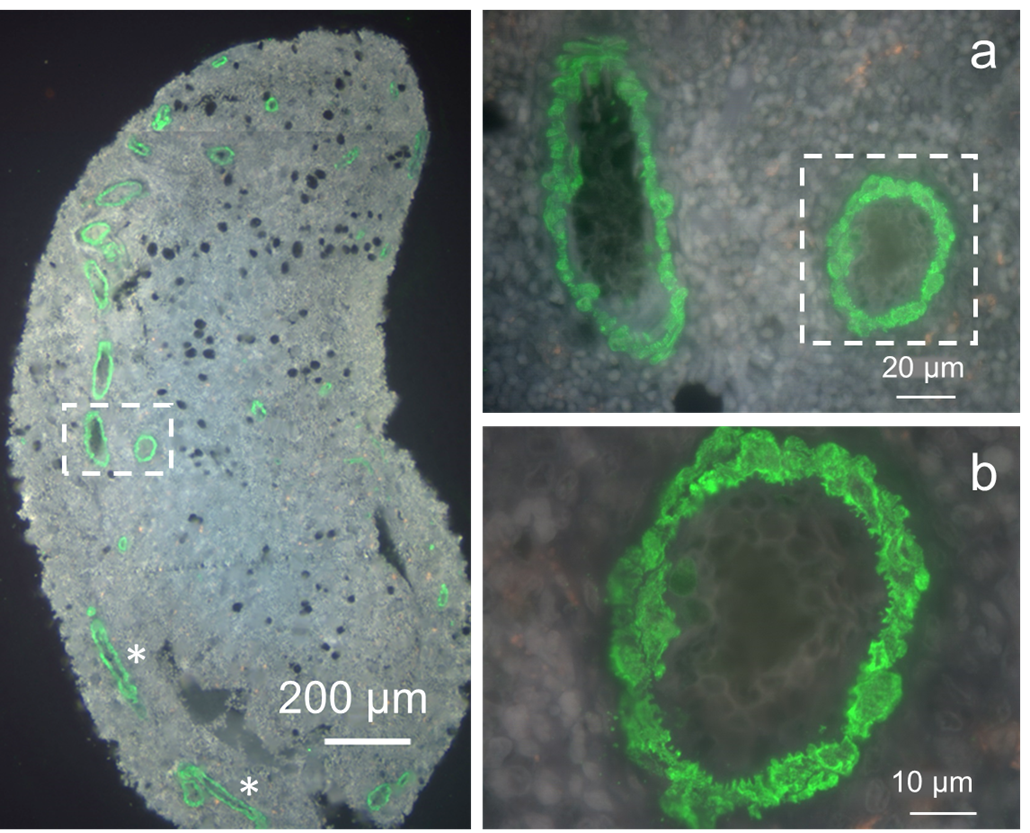


L

L

L

**Figure S9**. Fluorescence images of cells in the cross-sections of hemmule immunostained with anti-alpha actin antibody (left panel). The longitudinal sections of vessels are labeled with asterisks. The enlarged dashed area of the node is illustrated in panel **a**. An enlarged image of the transversal section of vessel of the panel **a** (dashed square is shown in panel b. L – Lumen.


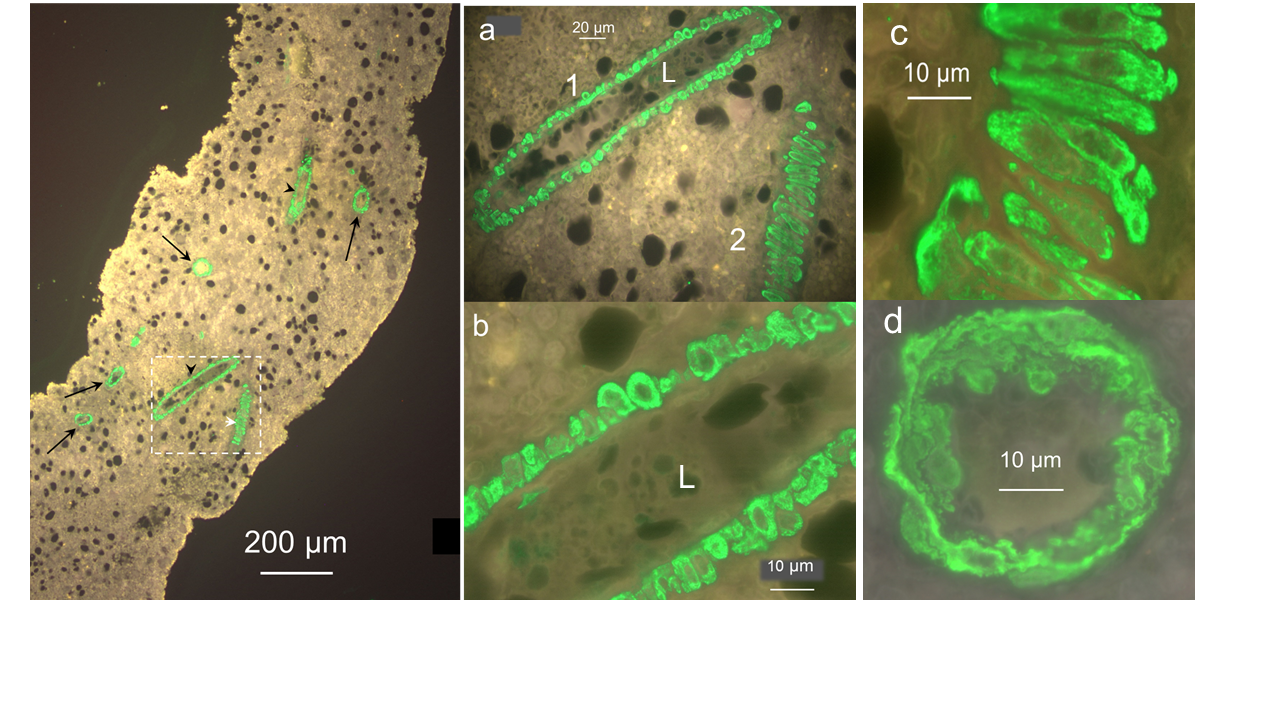


**Figure S10. Fluorescence images of cells in the cross-sections of hemmule immunostained with smooth muscle anti-alpha actin antibody**. Left panel: the longitudinal sections of vessels: arrowhead. The transversal sections of vessels: arrows. The enlarged dashed area of the hemmule is depicted in panel a. The enlarged transverse section of vessel 1 is shown in panel b. The portion of the wall labeled 2 at the single cell resolution is illustrated in panel c. d. The transveral section of a small vessel in bone marrow (positive control), L – lumen.

**
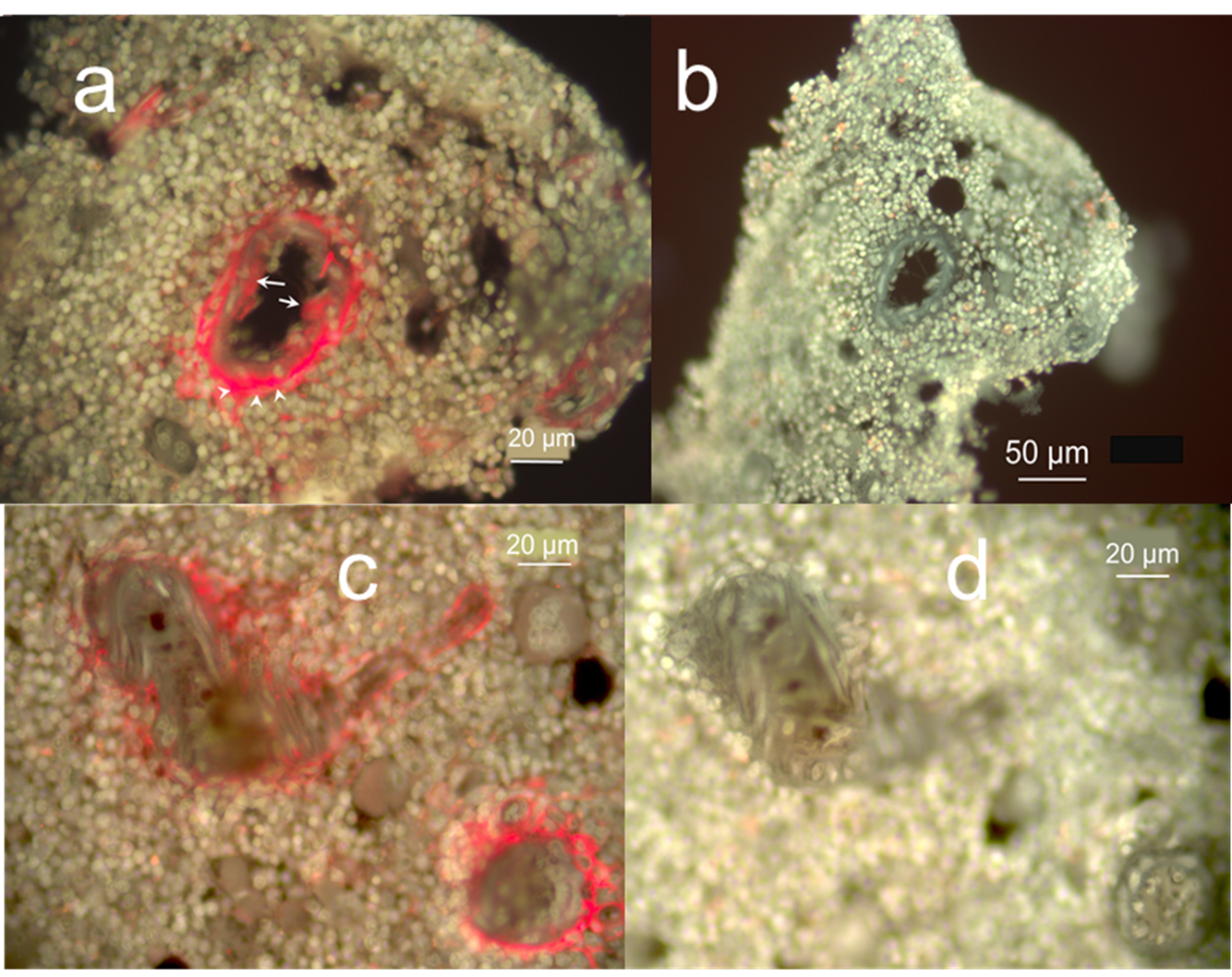
**

**Figure S11.** Fluorescence images of cells in the cross-sections of hemmule immunostained with anti-collagen antibody. **a.** Deposition of antibody along the transversal section of the node vessel shows fibers of the outmost external layer (Layer 1) of the vessel (arrowheads) and the internal endothelial cells (arrows) belonging to Layer 4. **b**. The section is shown in **a** with no antibody (control). **c**. The longitudinal (arrow) and transversal cross-section (arrowhead) of the vessel from area 2. The characteristic of the outmost external fibers around the vessel (Layer 1) are immunostained by the antibody. Layer 2 of smooth muscle cells is not stained. **d**. Control (no antibody). The longitudinal (arrow) and transversal cross-sections of the vessel are shown.


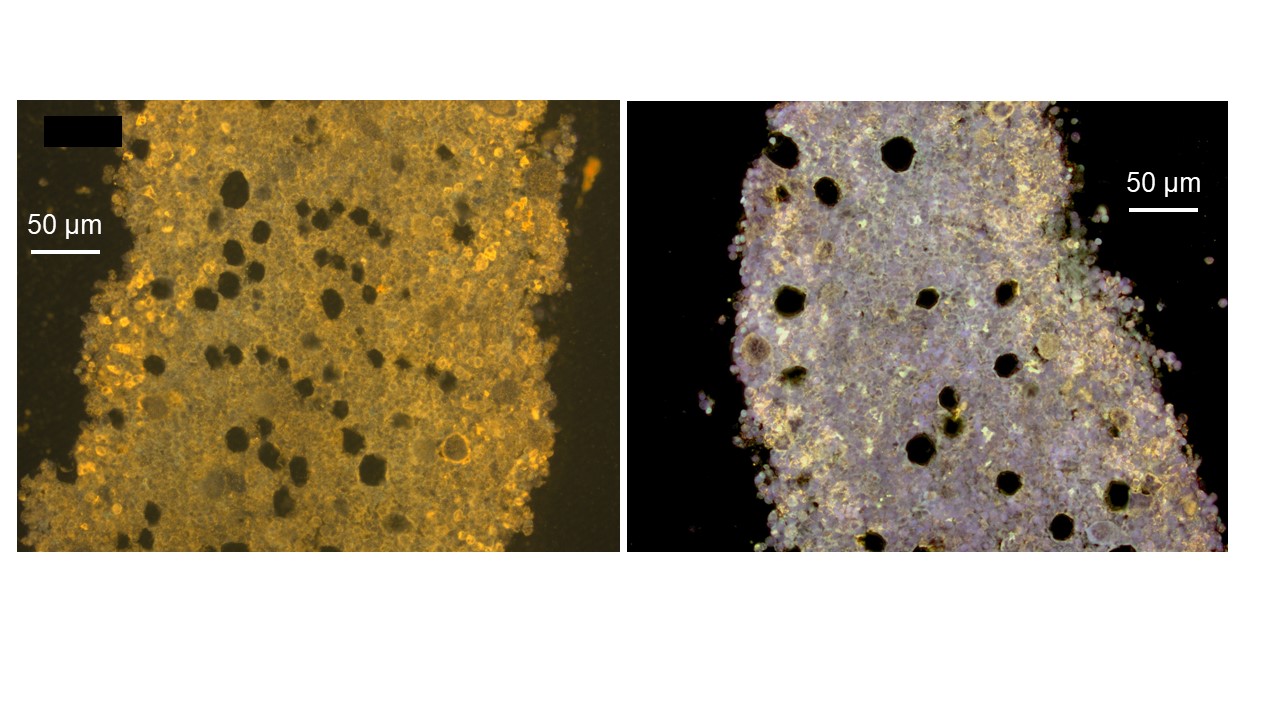


**Figure S12.** Fluorescence images of cells in the cross-sections of hemmule immunostained with anti OCT4 (left panel) and SOX2 (right panel) antibodies. The stained cell were distributed predominantly around the edges of the node.


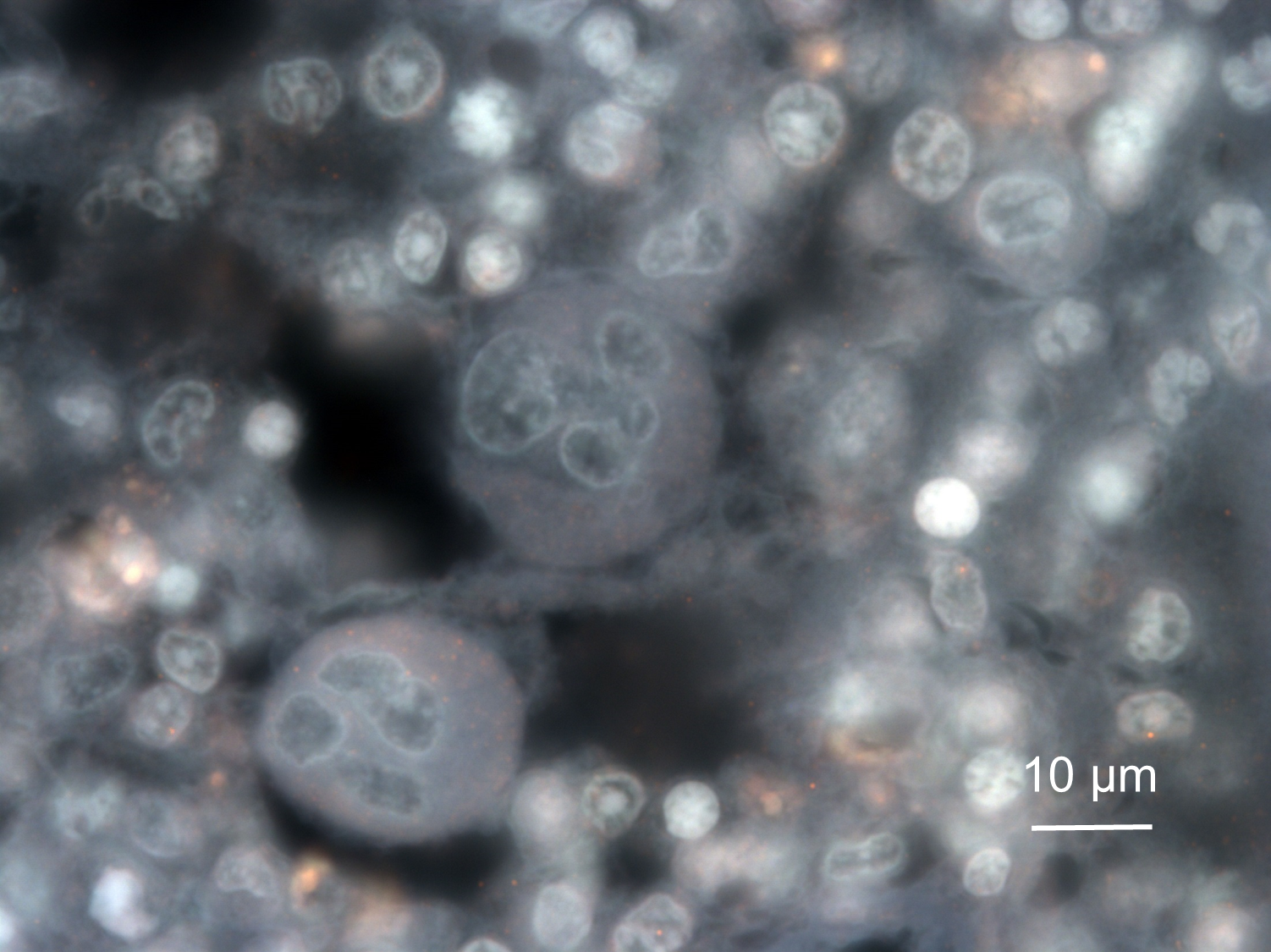


**Figure S13.** Large size cells in the bone marrow node.


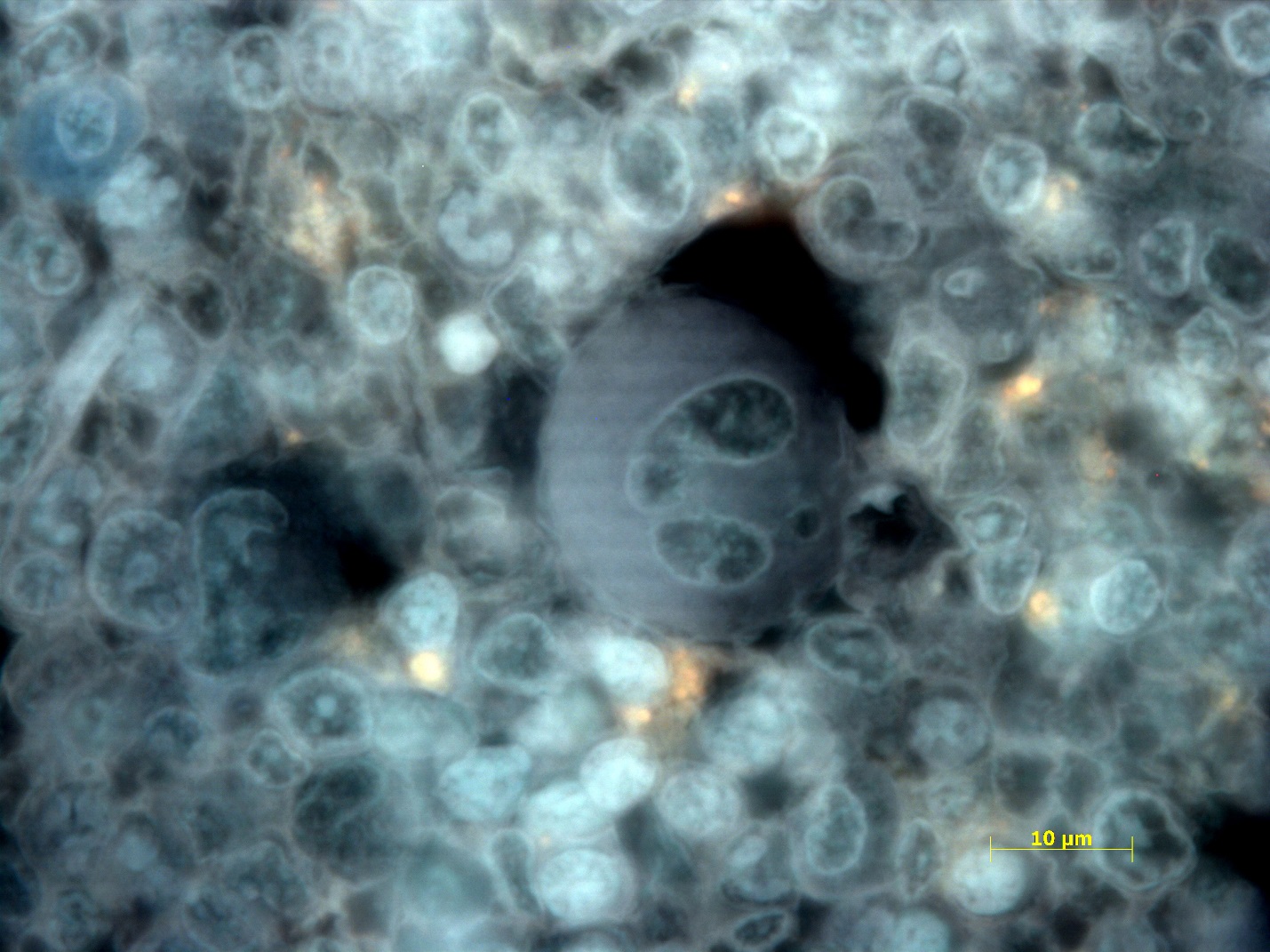


**Figure S14. L**arge size cell in the bone marrow node.


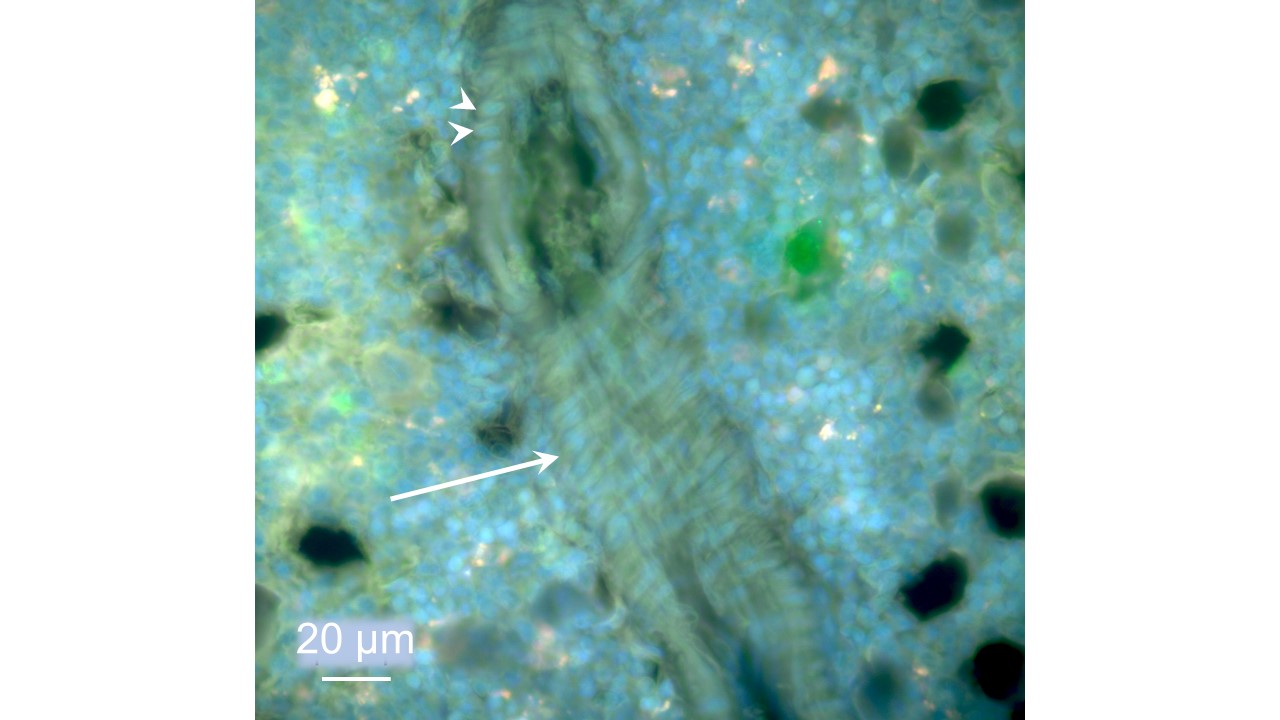


**Figure S15**. Fluorescence images of cells in the cross-sections of hemmule vessel immunostained with anti CD90 with DAPI. CD90 stains green while DAPI exhibits blue stain on nuclei of the same cells (arrowheads). The layer of smooth muscle cells shows a helix by CD90 green muscle cells with blue DAPI nuclei (arrow).

**S2 Table S4.** The RPKM levels of the osteogenic genes in BV, BM, LN, and H

| **Gene Symbol** | **Gene Name** | **BV** | **BM** | **LN** | **H** |
| --- | --- | --- | --- | --- | --- |
| Alpl | alkaline phosphatase | 3.995 | 3.62 | 0.139 | 122.613 |
| atf4 | activating transcription factor 4 | 74.859 | 189.74 | 251.219 | 92.493 |
| Bglap | bone gamma-carboxyglutamate protein | 274.854 | 423.603 | 0.078 | 71.813 |
| Bgn | biglycan | 42.053 | 33.478 | 5.673 | 45.618 |
| Bmp4 | bone morphogenetic protein 4 | 5.63 | 9.178 | 3.522 | 28.93 |
| Bmp6 | bone morphogenetic protein 6 | 3.363 | 3.177 | 0.024 | 22.069 |
| Col1a1 | collagen, type I, alpha 1 | 91.684 | 39.225 | 4.314 | 18.177 |
| Csf1 | colony stimulating factor 1 | 4.191 | 6.934 | 0.939 | 13.243 |
| Dcn | decorin | 13.774 | 10.611 | 6.563 | 13.02 |
| Igfbp3 | insulin-like growth factor binding protein 3 | 3.076 | 5.385 | 6.368 | 12.503 |
| Il18 | interleukin 18 | 10.156 | 20.469 | 17.146 | 11.241 |
| Jund | JunD proto-oncogene | 21.928 | 46.077 | 42.102 | 7.303 |
| Kitl | KIT ligand | 16.097 | 13.957 | 0.347 | 7.031 |
| Mmp13 | matrix metallopeptidase 13 | 13.455 | 12.146 | 0.003 | 5.674 |
| Nfatc1 | nuclear factor of activated T-cells 1 | 2.043 | 1.862 | 2.472 | 4.785 |
| Ogn | osteoglycin | 5.074 | 2.785 | 1.335 | 3.399 |
| Runx2 | runt-related transcription factor 2 | 1.45 | 3.47 | 0.214 | 2.972 |
| Sp7 | Sp7 transcription factor | 3.495 | 5.616 | 0.001 | 2.44 |
| Sparc | secreted protein acidic and cysteine rich | 181.107 | 122.517 | 12.757 | 2.333 |
| Spp1 | secreted phosphoprotein 1 | 32.069 | 16.623 | 0.464 | 2.09 |
| Thy1 | thymocyte nuclear protein 1 | 4.921 | 4.523 | 2.57 | 1.571 |
